# The genetic basis of cytoplasmic male sterility and fertility restoration in wheat

**DOI:** 10.1038/s41467-021-21225-0

**Published:** 2021-02-15

**Authors:** Joanna Melonek, Jorge Duarte, Jerome Martin, Laurent Beuf, Alain Murigneux, Pierrick Varenne, Jordi Comadran, Sebastien Specel, Sylvain Levadoux, Kalia Bernath-Levin, François Torney, Jean-Philippe Pichon, Pascual Perez, Ian Small

**Affiliations:** 1 ARC Centre of Excellence in Plant Energy Biology, School of Molecular Sciences, The University of Western Australia, Crawley, WA Australia; 2 Groupe Limagrain, Centre de Recherche, Route d’Ennezat, Chappes, France

**Keywords:** Agricultural genetics, RNA metabolism, Plant breeding, Plant hybridization

## Abstract

Hybrid wheat varieties give higher yields than conventional lines but are difficult to produce due to a lack of effective control of male fertility in breeding lines. One promising system involves the *Rf1* and *Rf3* genes that restore fertility of wheat plants carrying *Triticum timopheevii*-type cytoplasmic male sterility (T-CMS). Here, by genetic mapping and comparative sequence analyses, we identify *Rf1* and *Rf3* candidates that can restore normal pollen production in transgenic wheat plants carrying T-CMS. We show that Rf1 and Rf3 bind to the mitochondrial *orf279* transcript and induce cleavage, preventing expression of the CMS trait. The identification of restorer genes in wheat is an important step towards the development of hybrid wheat varieties based on a CMS-*Rf* system. The characterisation of their mode of action brings insights into the molecular basis of CMS and fertility restoration in plants.

## Abstract (web summary)

The development of hybrid wheat cultivars is hampered by the lack of an effective way to control male fertility in breeding lines. Here, the authors report the identification of two restorer-of-fertility genes *Rf1* and *Rf3* that can restore fertility of wheat plants carrying *Triticum timopheevii*-type cytoplasmic male sterility.

## Introduction

Genetic improvements in crop performance are crucial for increasing crop productivity, but current rates of improvement will not be sufficient to meet future food demands in an era of global climate change^1–3^. As arable land is limited, increased crop productivity must come from yield increases achieved by innovation in plant breeding^4–6^. By providing 20% of global dietary energy, wheat is one of the most important grain crops cultivated worldwide^7,8^, and together with maize and rice contributes 70% of global crop production^9^. However, while rice and maize yields have benefitted strongly from the use of hybrids to exploit heterosis, hybrid wheat varieties are not produced on a commercial scale, even though the estimated yield improvements could reach 20% in a context of climate change and increased disease pressure^10–12^. The lack of hybrid development has restricted the rate of wheat yield gain over the last two decades compared to yield gains in maize or rice^8,13^.

Hybrid production in autogamous plants requires a method to block self-pollination^14^. A system that has been used for production of hybrids in many crop plants including maize, rice and sorghum is based on cytoplasmic male sterility (CMS), a mitochondrially-encoded trait^15–17^, coupled with one or more nuclear *Restorer-of-fertility* (*Rf*) genes able to suppress CMS in F_1_ plants^14^. This breeding system exploits the genes controlling gynodioecy in natural populations of many flowering plants. Effective CMS sources have been discovered in wheat, such as T-CMS derived from a cross between *Triticum timopheevii* Zhuk. as female parent and bread wheat as the male parent^18^. However, a lack of effective *Rf* genes has been a major factor limiting the application of CMS to hybrid seed production in wheat^13^.

Based on studies in other plant species, it is known that Rf proteins are encoded in the nucleus and post-translationally imported to mitochondria, where they generally prevent the accumulation of gene products from CMS-specific open reading frames (ORFs)^17,19,20^. The majority of *Rf* genes in higher plants encode RNA-binding pentatricopeptide repeat (PPR) proteins^14,21,22^. The wheat genome contains a surprisingly large number (over 200) of *Restorer-of-fertility-like* (*RFL*) genes, the majority of which are organised in clusters on chromosomes 1, 2 and 6^23^. Several *Rf* genes controlling fertility in the T-CMS system have been reported in *Triticum aestivum* L. that map to these RFL clusters, including *Rf1* (chr1A)^24–27^ and *Rf3* (chr1B)^28–33^.

Here, we report the identification of Rf1 and Rf3 as PPR proteins and demonstrate their ability to suppress T-CMS in wheat by transgenesis. Both proteins bind to and induce cleavage of transcripts of a previously unrecognised mitochondrial gene (*orf279*) with no impact on the processing of *orf256*, the gene previously thought to cause male sterility in wheat.

## Results

### Mapping of the genomic regions harbouring *Rf1* and *Rf3* restorer genes

**Fig. 1:**
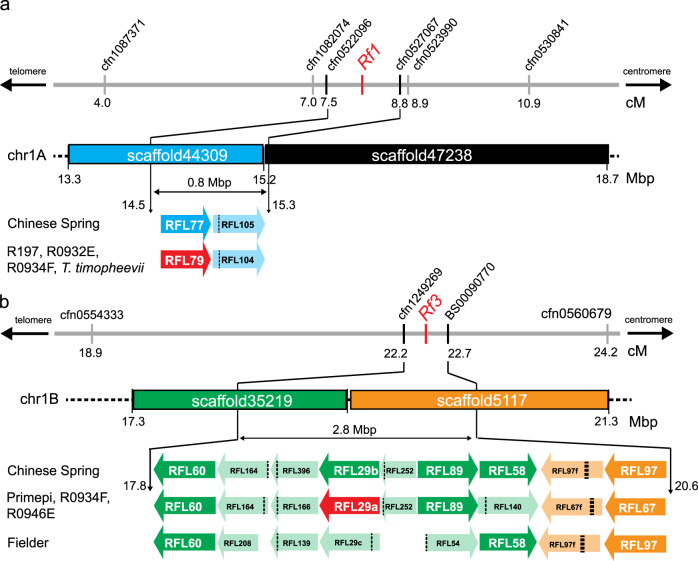
Genetic map of the *Rf1* and *Rf3* loci in wheat Chinese Spring. **a** The genetic markers delimiting the *Rf1* interval on chromosome 1A (chr1A) were genotyped and mapped in three populations segregating for male-fertility restoration conferred by the *Rf1* gene. The 0.8 Mbp region delimited by marker cfn0522096 and cfn0527067 is indicated and the RFL genes present in the interval are shown. **b** The genetic markers delimiting the *Rf3* interval on chromosome 1B (chr1B) were genotyped and mapped in three populations segregating for *Rf3* and a doubled-haploid line carrying the *Rf3* gene. The 2.8 Mbp region delimited by marker cfn1249269 and BS00090770 is indicated and the RFL genes present in the interval are shown. Scaffold sequences were obtained from the URGI IWGSC sequence repository (https://wheat-urgi.versailles.inra.fr/). Gene sequences that are truncated or disrupted by a frameshift are indicated by lighter colouring. The proposed order of the genes in the *Rf3* interval in the *Rf3* and Fielder line is only approximate. cM centimorgan, Mbp megabase pair. The marker sequences are listed in Supplementary Table 1.

We analysed three F_2_ mapping populations (R197xKalahari, R204xAlixan and R0932ExAltigo) segregating for *Rf1* and three F_2_ mapping populations (TJB155xAnapurna, 2852xAltamira, and AH46xR0946E) segregating for *Rf3*. Fine mapping by analysis of the progenies of recombinant plants defined the *Rf1* interval to be a region between 7.5 and 8.8 cM on chromosome 1A (Fig. 1a), and the *Rf3* interval to be between 22.2 and 22.7 cM on chromosome 1B (Fig. 1b). The International Wheat Genome Sequencing Consortium (IWGSC) RefSeq v1.0 assembly^23^ was used to anchor these intervals to genome sequence scaffolds (Fig. 1).

### RFL genes present in the *Rf1* and *Rf3* intervals in the wheat reference genome

In the IWGSC RefSeq v1.0 reference genome^23^, 13 genes are annotated in the *Rf1* interval of which two are RFL-type PPR genes. In the slightly larger *Rf3* interval, 28 genes are present, nine of which are RFL-type PPR genes (Fig. 1, Supplementary Table 2). Of the two RFL genes identified in the *Rf1* interval on chromosome 1A, only one encodes a full-length PPR protein; the other is truncated at the C-terminus (Fig. 1a, Supplementary Table 2). The *Rf3* interval on chromosome 1B includes five full-length RFL genes, three truncated RFL genes and one frame-shifted RFL open reading frame (Fig. 1b, Supplementary Table 2). The full-length RFL sequences present in the intervals are composed of 741–790 codons and 17–19 PPR motifs (Supplementary Table 2). TargetP v1.01^34^ identified a mitochondrial targeting peptide (mTP) (Supplementary Table 2) at the N-terminus of the majority of the proteins.

### Enrichment and sequencing of RFL genes in *T. timopheevii* and eight wheat accessions

The *T. aestivum* Chinese Spring accession from which the reference genome was obtained is classified as a partially restoring genotype containing a weak *Rf3* allele^35^. To identify *Rf1* and *Rf3* candidates from restoring genotypes without the need for full genome sequencing and assembly, a customised RFL capture approach was developed. DNA baits were designed based on 1199 RFL (PPR) sequences extracted from 33 genomic and two transcriptome data sets from 27 cereal species and their wild relatives (Supplementary Table 3). These baits were used to enrich genomic DNA fragments containing RFL sequences from eight wheat accessions and *T. timopheevii* (Supplementary Table 4). The selected DNA fragments were sequenced on an Illumina MiSeq platform. On average, 220 RFL contigs were obtained from each hexaploid *T. aestivum* accession and 138 from the tetraploid *T. timopheevii* (Supplementary Table 4). For each of the 216 RFL sequences in the Chinese Spring reference genome, a corresponding sequence was obtained by the RFL capture approach with the only differences being due to occasional incomplete assembly of the full reading frame outside of the PPR motifs (Supplementary Table 4), confirming the accuracy of the assembly approach used for the captured fragments. Within these contigs, RFL-type ORFs were identified across all nine genotypes. The number of ORFs per accession ranged from 143 in *T. timopheevii* to 262 in accession R0946E (Supplementary Table 4). To identify putatively orthologous RFL sequences, they were grouped using CD-HIT v4.6.4^36^ using a threshold of 96% sequence identity (Supplementary Data 1), which we found to be the best compromise between distinguishing paralogous sequences within each accession while grouping putatively orthologous sequences across accessions.

### Selection of candidate *Rf1* and *Rf3* orthologous groups

We selected candidate *Rf1* and *Rf3* groups from the 397 orthologous RFL groups based on whether the restoring genotypes contained a putatively full-length, functional member of that group, while the non-restoring genotypes did not. The list of candidates was refined by excluding those that mapped outside the intervals established by the genetic mapping (Supplementary Table 5). In addition, as the *Rf1* gene most likely originates from *T. timopheevii*^24,25^, the *Rf1* candidate groups should contain a representative from *T. timopheevii*. On the contrary, *Rf3* originates from *T. aestivum*^33^, therefore the candidate *Rf3* groups should not contain an identical representative from *T. timopheevii*.

First, we identified the orthologous RFL groups comprising at least one sequence captured from an accession characterized as carrying the *Rf1* gene (accessions R197, R0932E, R0934F and *T. timopheevii*) (Supplementary Table 5). In silico mapping of the candidate sequences to estimate their genomic location on chromosome 1A was achieved by aligning the DNA contigs to the reference genome. To confirm the location of the candidate RFLs within the *Rf1* interval, several gene-specific markers were developed for each gene and genetic mapping was performed (Supplementary Table 5). The mapping allowed orthologous RFL groups that mapped outside of the *Rf1* interval on chromosome 1A (Supplementary Table 5) to be discarded. In this way, four orthologous RFL groups (79, 104, 185 and 268) were identified as potential candidates for the *Rf1* gene in wheat (Supplementary Table 5). The protein sequences from groups RFL185 and RFL268 are only partial (<500 amino acids long) and correspond to two parts of the same gene that was split by a frameshift (Supplementary Data 1). Therefore, both proteins were considered as unlikely to be functional and excluded as candidates for *Rf1*. As both RFL79 and RFL104 are composed of 750+ amino acids and ~17 PPR motifs (Supplementary Table 5) these two sequences were considered as being the best candidate *Rf1* groups.

To select candidates for *Rf3*, orthologous RFL groups were screened for full-length protein sequences present in the *Rf3*-containing Primepi, R0946E and R0934F genotypes but absent from (or partial in) the *rf3* genotypes. This analysis allowed the identification of nine candidate groups of which seven were identified to be located within the *Rf3* interval by genetic mapping (Supplementary Table 5). *RFL29* and *RFL89* orthogroups encode full-length RFL proteins (Supplementary Table 5). Three versions of *RFL29* exist within the *RFL29* group (Fig. 1b, Supplementary Fig. 1). The *RFL29a* allele is present in Primepi, R0946E and R0934F, the *RFL29b* allele is found in the weak restorer line Chinese Spring, and the *RFL29c* version found in Fielder carries an indel that disrupts the coding sequence (Fig. 1b, Supplementary Fig. 1). RFL29a and RFL29b are highly similar, with only eight amino acid polymorphisms and two extra amino acids inserted near the N-terminus of RFL29b (Supplementary Fig. 1). There is also an insertion in the putative 5′ untranslated region (UTR) of *RFL29b* (Supplementary Fig. 1). This, in addition to the coding sequence polymorphism observed between *Rf3* lines and non-restoring accessions, indicated *RFL29a* as a candidate for *Rf3*. The sequences from *Rf3* genotypes Primepi, R0946E and R0934F appear to be full-length and putatively functional (Supplementary Table 5). In groups *RFL67* and *RFL89*, the only full-length sequences identified were from *Rf3* genotypes.

### Expression of *Rf1* and *Rf3* candidates in anthers during pollen formation

**Fig. 2:**
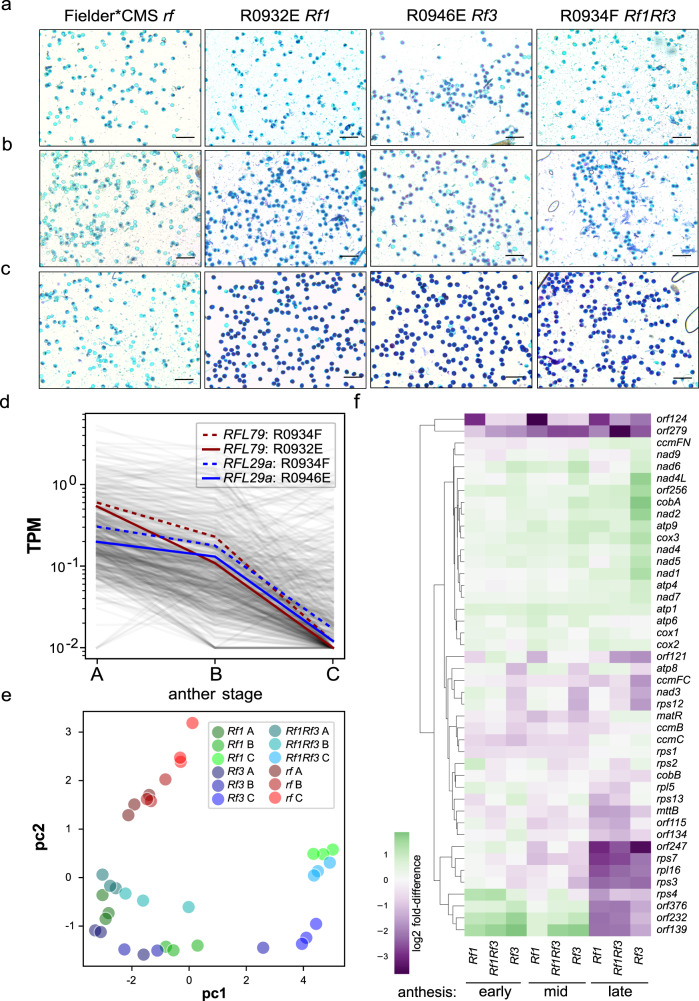
Expression profiles of nuclear and mitochondrial genes in fertile and sterile wheat genotypes during anther development. Alexander’s staining of viable and non-viable pollen grains from anthers collected at three developmental stages: early heading (**a**), early/mid anthesis (**b**) and late anthesis (**c**). Scale bar = 200 μm. This experiment was performed twice. A representative from 20 micrographs for each genotype is shown. **d** Expression profile of *RFL* genes in three wheat genotypes: R0932E carrying *Rf1*, R0946E carrying *Rf3* and R0934F carrying *Rf1* and *Rf3*. The expression profiles of *RFL79* and *RFL29a*—the two candidates for *Rf1* and *Rf3*—are highlighted in blue and red, respectively. TPM: Transcript per million values calculated from RNA-Seq data. Anther stages A, B and C correspond to the three developmental stages of anther development as described in (**a**–**c**). **e** Principal components analysis (PCA) of the read counts for 44 mitochondrial transcripts. The first principal component (pc1, 65% of variance) distinguishes between the three stages in the fertile genotypes and the second principal component (pc2, 14% of variance) distinguishes the fertile and sterile genotypes. **f** Hierarchical clustering of 44 mitochondrial transcripts based on read counts in fertile lines relative to the sterile line at the equivalent developmental stage calculated from RNA-Seq data. *Rf1* = R0932E, *Rf3* = R0946E, *Rf1Rf3* = R0934F. Early, mid and late anthesis correspond to the three developmental stages of anther development as described in (**a**–**c**). This experiment was performed once. Three biological replicates each comprising 15 anthers from 3 to 4 individual spikelets were used for RNA extraction and the expression analysis. Source data underlying Fig. 2a–c are provided as a Source data file. Data and code used to generate the plots in Fig. 2d–f are obtainable from Dryad^88^.

We looked at gene expression during anther development to see if these *Rf1* and *Rf3* candidates are expressed during the critical phases when differences between male-sterile and male-fertile genotypes become apparent. Three stages of anther development were chosen as visualized by pollen staining in Fig. 2a–c, and corresponding to early, mid and late anthesis respectively. Based on the use of Alexander’s stain to distinguish viable and non-viable pollen, clear differences between sterile and fertile plants are visible at mid and fully developed by late anthesis (Fig. 2b, c). Total RNA was extracted from developing anthers at these three stages, depleted of rRNA and sequenced using random hexamer primers to allow unbiased capture of mitochondrial transcripts in addition to cytosolic and nuclear transcripts. Principal components analysis (PCA) of the read counts for each transcript showed strong differentiation between the three stages in the fertile plants (first principal component, Supplementary Fig. 2) and strong differentiation between the fertile and sterile genotypes (second principal component, Supplementary Fig. 2). The RNA-Seq reads were mapped against the captured RFL contigs for each line to determine expression levels for each RFL gene. Most RFL genes are expressed at low levels (0.1–1 transcript per million) at early anthesis (anther stage A), with the expression level falling by mid anthesis (anther stage B) and often undetectable by late anthesis (anther stage C) (Fig. 2d). The *Rf1* and *Rf3* candidates follow this pattern, although with expression levels a little above the median of RFL expression, particularly at mid anthesis (anther stage B). Thus, the *Rf1* and *Rf3* candidates are expressed in anther tissue in the critical period when pollen formation starts to differ between fertile and sterile lines.

As RFL proteins are expected to have an impact on mitochondrial gene expression, we analysed mitochondrial transcripts in the same RNA-Seq experiment, including not only all the known coding sequences but also ten open reading frames exceeding 100 codons that are only found in the *T. timopheevii* mitochondrial genome and that could putatively encode CMS-specific polypeptides. Remarkably, PCA analysis on just the 44 mitochondrial transcripts (Fig. 2e) largely recapitulated the pattern found with 130,404 nuclear and cytosolic transcripts (Supplementary Fig. 2), showing the strong correlation between mitochondrial gene expression during anther development and male fertility. Hierarchical clustering of mitochondrial transcripts based on read counts in fertile lines relative to the sterile line (Fig. 2f) showed a large cluster encoding components of the respiratory chain (complexes I, III, IV and V) that accumulate in the male-fertile lines. In contrast, transcripts for components of the mitochondrial biogenesis machinery (e.g. ribosomal subunits, cytochrome *c* maturation system) tend to be lower in the fertile lines, particularly by late anthesis. Of the *T. timopheevii*-specific ORFs, most show lower expression in the fertile lines. The surprising exception is *orf256*, whose expression was previously reported to be correlated with T-CMS in wheat^37,38^. In contrast, expression of two other T*. timopheevii*-specific ORFs (*orf124* and *orf279*) is strongly suppressed in the fertile lines.

### Testing the restoring capabilities of *Rf1* and *Rf3* candidate genes by transgenesis

**Fig. 3:**
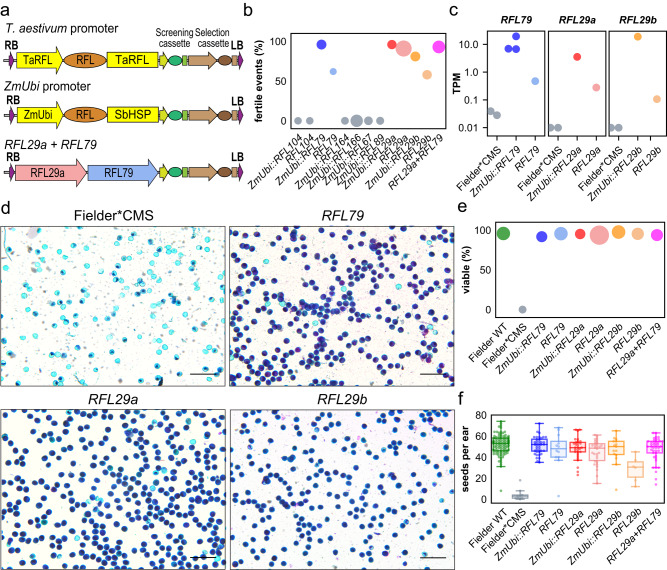
Transformation of *Rf1* and *Rf3* candidate genes into the Fielder*CMS line. **a** Design of the constructs used in plant transformation experiments. *TaRFL*, sequence from the wheat *Rf* candidate gene; *ZmUbi*, *Zea mays* ubiquitin promoter; *SbHSP*, 3′-UTR from *Sorghum bicolor* heat shock protein gene. The screening cassette consists of the *T. aestivum* HMWG gene promoter, *ZsGreen* coding sequence and *tNos* terminator sequence. The selection cassette consists of a rice actin gene promoter, the *bialaphos resistance* (*Bar*) gene and *tNos* terminator sequence. RB right border, LB left border. **b** Evaluation of fertility restoration by *Rf1* and *Rf3* candidates based on the percentage of T_0_ events giving fertile plants. The area of the plot markers is proportional to the total number of transformation events obtained, ranging from 13 to 69. **c** Expression level of the restoring transgene in transcript per million (TPM) values calculated from RNA-Seq data. The values shown are TPM + 0.01 to allow the Fielder*CMS results to be plotted on a log scale; all three of these genes are absent from Fielder and undetected in the cases of the *RFL29a* and *RFL29b*; a few reads mapped to *RFL79* indicating a low level of cross-mapping from related RFL genes. **d** Alexander’s stain of pollen grains collected from anthers at late anthesis (anther stage C) of Fielder*CMS and restored transformants (T_1_ generation). This experiment was performed once. The selected micrographs are representatives of 10–15 individual images. Scale bar = 200 μm. **e** Pollen viability counts from analysis of 10–15 images like those in (**d**). The area of the plot markers is proportional to the number of pollen grains counted, ranging from 920 to 5215. **f** Plant fertility based on seed set per ear on T_1_ plants from five different transgenic events per construct. Number of ears analysed per line: Fielder WT *n* = 1043, Fielder*CMS *n* = 125, *ZmUbi::RFL79* *n* = 313, *RFL79* *n* = 92*, ZmUbi::RFL29a* *n* = 209*, RFL29a* *n* = 197*, ZmUbi::RFL29b* *n* = 107*, RFL29b* *n* = 43*, RFL29a* + *RFL79* *n* = 359. Centre line, median; box limits, upper and lower quartiles; whiskers, 1.5× interquartile range. Source data underlying (**b**) and (**d**–**f**) are provided as a Source data file. Data and code used to generate the plots in panel Fig. 3c are obtainable from Dryad^88^.

The coding sequences of *RFL79* and *RFL104* (*Rf1* candidates) as well as of *RFL29a/b*, *RFL67*, *RFL164*, *RFL166* and *RFL89* (*Rf3* candidates) were cloned into plant transformation vectors under the control of the *Zea mays* ubiquitin promoter (*ZmUbi*). The *RFL104*, *RFL79* and *RFL29a/b* genes were also cloned under the control of their own promoter (Fig. 3a). The constructs were transformed into a specifically developed Fielder*CMS wheat line—a cultivar harbouring *T. timopheevii* cytoplasm (T-CMS) but a *T. aestivum* nuclear genome (‘Fielder’ background) showing high efficiency for *Agrobacterium tumefaciens* transformation^39^. Transgene insertion in the genome was confirmed by PCR (Supplementary Data 2) and the fertility of T_0_ transgenic plants was evaluated. No pollen was observed to be produced and no seed set by Fielder*CMS plants or Fielder*CMS plants transformed with *RFL104*, *RFL164*, *RFL166*, *RFL89* or *RFL67* (Fig. 3b, Supplementary Fig. 3). In contrast, fertility was observed in many plants expressing *RFL79* or *RFL29a/b* sequences driven by either their own promoters or the *ZmUbi* promoter. Fertility restoration was quantitatively evaluated using the frequency of transformation events leading to fertility vs. sterility for each construct (Fig. 3b). Fertility restoration observed in the T_0_ generation for the *RFL79* transgene was 96% (*ZmUbi* promoter) and 62% (endogenous promoter), respectively. Similarly, the two *Rf3* candidates, *RFL29a* and *RFL29b*, were observed to confer strong fertility restoration (over 80%) with the *ZmUbi* promoter. With their endogenous promoters, *RFL29a* shows better fertility penetrance than *RFL29b* (Fig. 3b), in line with a higher level of expression as evaluated by RNA-Seq (Fig. 3c). A construct combining *RFL79* and *RFL29a* under the control of their own regulatory sequences gave 93% of events showing fertility restoration. Male fertility was investigated more thoroughly in the subsequent generation by staining pollen for viability (Fig. 3d, e) and counting seeds set per ear and per spikelet (Fig. 3f, Supplementary Fig. 3b).

**Fig. 4:**
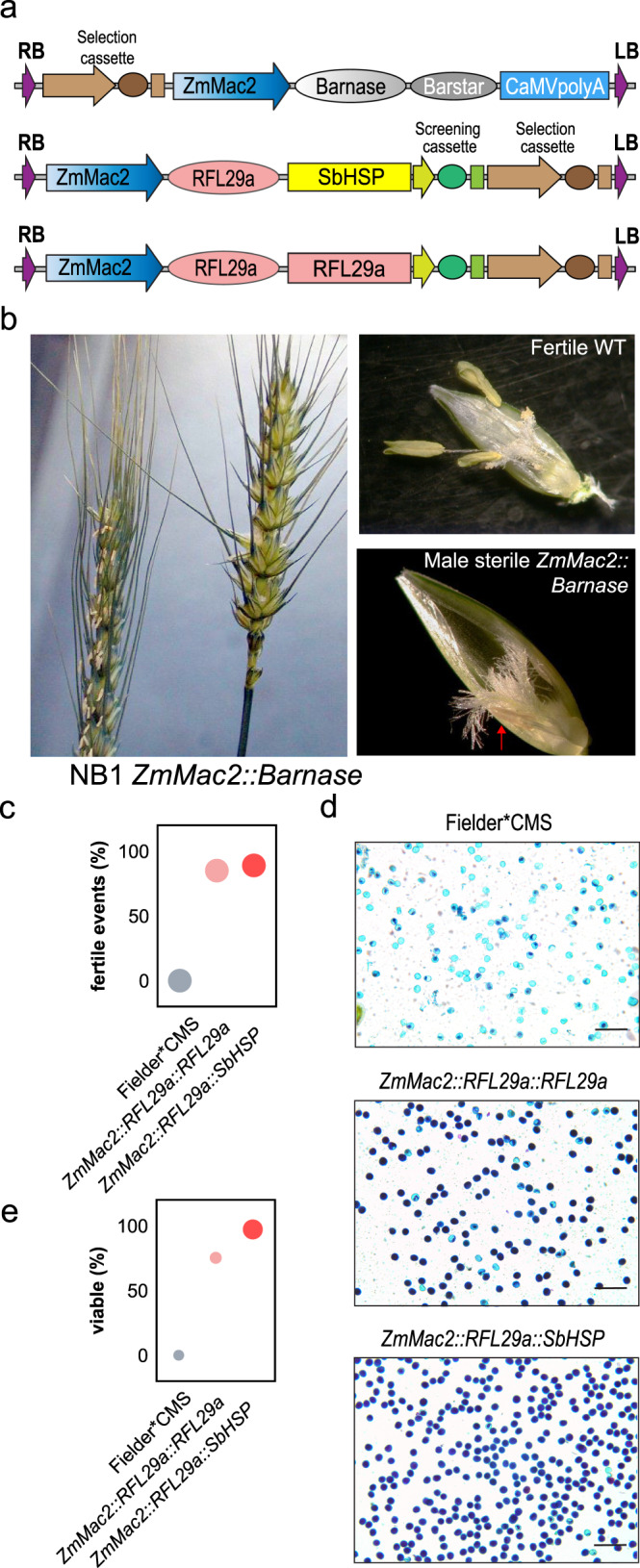
Restoration of fertility by *RFL29a* driven by a tapetum-specific promoter. **a** Constructs used in the experiment. **b** Images showing complete male sterility induced by expression of the *ZmMac2::Barnase* construct in wheat. Red arrow shows aborted anther with only filament. **c** Plant fertility is restored by expression of *ZmMac2::RFL29a*. Plot marker area is proportional to the total number of transformation events obtained, ranging from 19 to 20. **d** Pollen from *ZmMac2::RFL29a* plants stained for viability, showing substantial restoration of normal pollen development. This experiment was performed once. A representative from 20 micrographs is shown. Scale bar = 200 μm. **e** Pollen viability counts. Plot marker areas are proportional to the number of grains counted, ranging from 920 to 3142. Source data underlying Fig. 4b–e are provided as a Source data file.

That these genes act through a role in anther development and pollen formation was tested by attempting to restore fertility using *RFL29a* driven by a tapetum-specific promoter, *ZmMac2* (Fig. 4a). In maize anthers, this promoter is active in the tapetal cell layer during the pollen tetrad stage, with decreasing activity as free microspores are released^40^. Its expression pattern is likely to be similar in wheat, as when used to drive expression of cytotoxic barnase it specifically causes male-sterility, without affecting vegetative or female organ development (Fig. 4b). Two constructs using *ZmMac2* to drive *RFL29a* expression substantially restored male fertility and pollen formation when used to transform Fielder*CMS (Fig. 4c–e).

### *orf279* is the cause of T-CMS in wheat

Previous studies indicated that fertility restoration of T-CMS plants is linked with the expression of Orf256 protein^37^ and that the nuclear background influences the processing of the *orf256* transcript in wheat accessions^38^. Thus, the expression of *orf256* was analysed in *RFL79* and *RFL29a* transformants by RT-PCR (Supplementary Fig. 4). The *orf256* transcript is detected in both CMS and restored lines (Fig. 2f) and northern blot analysis revealed that partial cleavage of *orf256* RNA already occurs in the sterile Fielder*CMS plants and no additional *orf256* cleavage is observed in the fertile Fielder*CMS plants transformed with the *ZmUbi::TaRFL79* or *ZmUbi::TaRFL29a* constructs (Supplementary Fig. 4). 5′-Rapid Amplification of cDNA Ends (5′-RACE) analysis confirmed that the cleavage of *orf25*6 does not correlate with the restoration of fertility phenotype observed in the transformants (Supplementary Fig. 4). Furthermore, northern blot analysis of several wheat accessions carrying T-CMS cytoplasm showed no correlation between the processing of *orf256* and the presence of either *Rf1* or *Rf3*, and cleavage of *orf256* is also detected in sterile genotypes (Supplementary Fig. 4d).

**Fig. 5:**
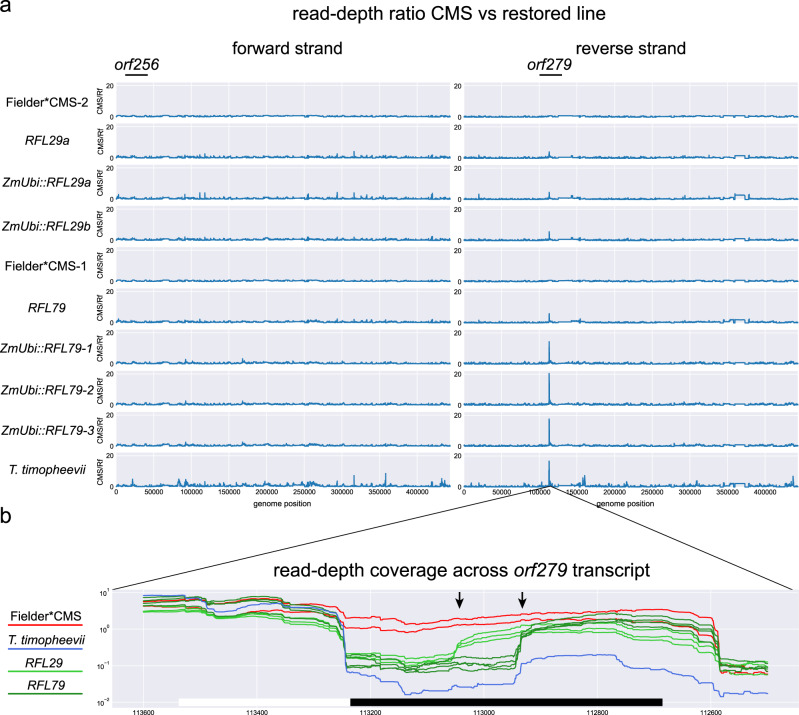
Identification of *orf279* as a gene associated with T-CMS in wheat. **a** Ratio of strand-specific RNA-Seq coverage from Fielder*CMS lines (sterile) and restored (fertile) samples plotted across the *T. timopheevii* mitochondrial genome (NCBI accession number NC_022714.1) for forward and reverse strand is shown. The genomic regions carrying *orf256* (forward strand) and *orf279* (reverse strand) are indicated at the top of the plots. The number of replicates for *ZmUbi::RFL29a*, *ZmUbi::RFL29b, RFL29a*, *RFL79* and *T. timopheevii* was *n* = 1, Fielder*CMS *n* = 2 and for *ZmUbi::RFL79* *n* = 3. **b** Normalised RNA-Seq coverage in the *orf279* region. *orf279* is indicated by the boxes below the chart, distinguishing the part of *orf279* that is identical to *atp8* (white box) and the *orf279*-unique region (black box). The number of RNA-Seq reads mapped to the central region of *orf279* in restorer line is much lower than in Fielder*CMS. The sharp transition from low to high coverage (arrows) indicates the probable site of RNA cleavage induced by a restorer gene. Data and code used to generate the plots in the panels are obtainable from Dryad^88^.

**Fig. 6:**
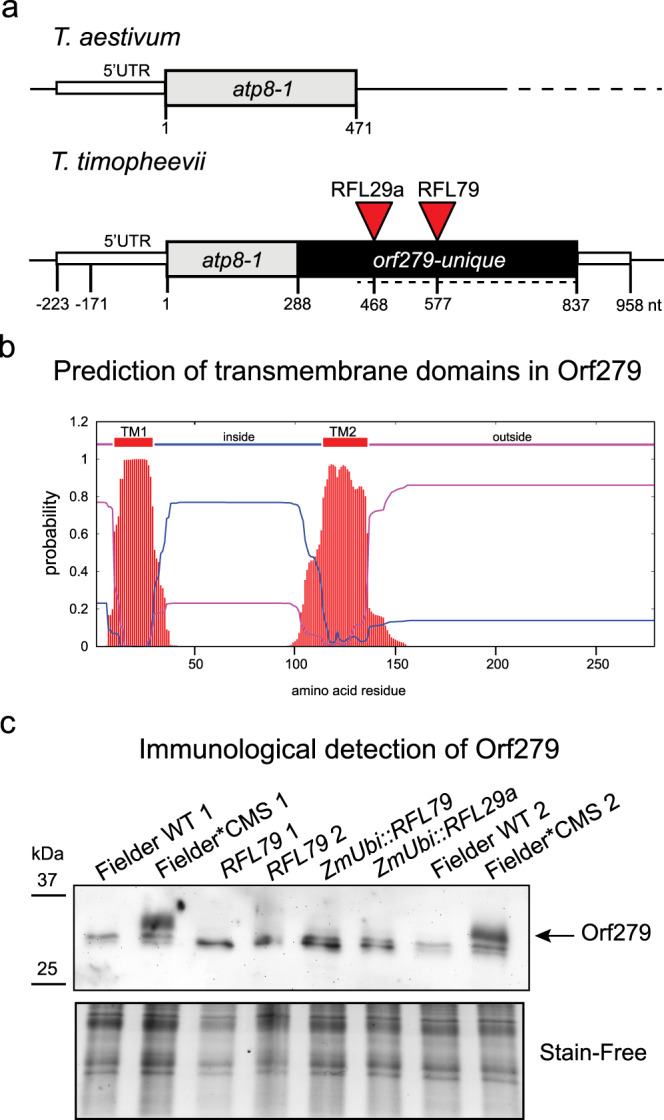
Characterisation of *orf279* as genetic basis of T-CMS in wheat. **a** Comparison of the *atp8-1* gene from *T. aestivum* and *orf279* gene from *T. timopheevii* genome. The gene coding sequences are shown as grey and black boxes. The 5′ and 3′ regions are indicated. The part of *orf279* used for production of the Orf279 antibody is indicated by a dashed line. The RNA cleavage sites induced by RFL29a and RFL79, the two candidates for Rf3 and Rf1 restorer in wheat, are shown, and were estimated based on RNA-Seq and 5′ RACE results shown in Supplementary Fig. 6. **b** Two transmembrane domains in the Orf279 protein sequence were predicted with TMHMM method^41^ on the TMHMM Server v. 2.0 (http://www.cbs.dtu.dk/services/TMHMM/) and are shown in red. TM1 (transmembrane 1) and TM2 (transmembrane 2) encompass amino acid residues 10–29 and 114–136, respectively. TM1 is located within the Atp8 region of Orf279. **c** Immunological detection of Orf279 in mitochondrial protein extracts. Mitochondrial fractions were enriched from 11-day-old etiolated seedlings grown on vermiculite, treated with n-dodecyl-β-d-maltoside to release membrane-bound proteins and separated on a 15% Stain-Free™ SDS-PAGE gel (Bio-Rad). A Stain-Free image of the gel is shown as a loading control. This experiment was performed three times with similar results. Source data underlying Fig. 6c are provided as a Source data file.

As *orf256* did not appear to be related to fertility restoration in our material, we carried out a systematic RNA-Seq analysis of RNA samples from Fielder*CMS and restored lines. We observed no significant differences in expression of *orf256* (Fig. 5a) or *orf124* (Supplementary Fig. 5) but did notice major differences in the expression of a previously unrecognised gene, which we designate *orf279* (as the reading frame consists of 279 codons). The 5′ flanking sequence and the first 96 codons of *orf279* are identical to *atp8* (Fig. 5b). The remaining 184 codons are contained within a 552 nt sequence present in the *T. timopheevii* genome but absent from all other sequenced mitochondrial genomes, including the *T. aestivum* mitochondrial genome (Fig. 5b). RNA-Seq coverage within part of this unique sequence is lower within the restored plants than in sterile plants. The coverage pattern is consistent with cleavage of *orf279* transcripts within the unique region followed by degradation of the 5′ cleavage product and persistence of the 3′ cleavage product (Fig. 5b). The RNA-Seq data show that the putative 3′ cleavage product is ~110 nt longer in *Rf3* plants than in *Rf1* plants. This differential processing of *orf279* transcripts in *RFL79* and *RFL29a* lines was confirmed by RACE (Supplementary Fig. 6). A major 5′-RACE product of ~205 nt was detected in the *RFL79* transformants, whereas in *RFL29* transformants a major amplicon of ~310 nt was found (Fig. 6a, Supplementary Fig. 6). In agreement with the provenance of *Rf1* from *T. timopheevii* and *Rf3* from *T. aestivum*, only the *Rf1*-specific 5′-RACE product was detected in *T. timopheevii* (Supplementary Fig. 6).

Two transmembrane domains were predicted in the putative *orf279* gene product with the TMHMM method^41^ (Fig. 6b). TM1 (transmembrane 1) and TM2 (transmembrane 2) encompass amino acid residues 10–29 (within the Atp8 region) and 114–136 (within the unique region), respectively. A polyclonal antibody directed towards a recombinant protein encompassing the unique part of Orf279 detected a protein of 32 kDa in a mitochondrial protein fraction extracted from Fielder*CMS seedlings but not in equivalent fractions from Fielder or lines transformed with *RFL29* or *RFL79* (Fig. 6c).

### RFL79 and RFL29a bind to the *orf279* transcript in vitro

**Fig. 7:**
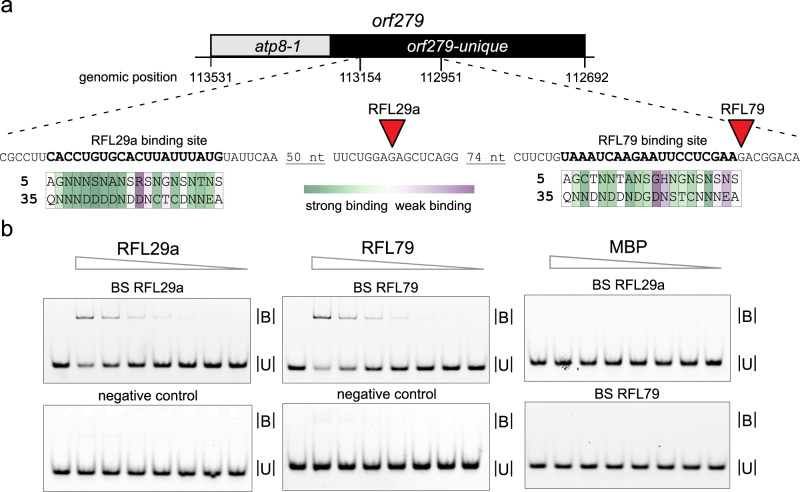
RNA-binding assays with recombinant RFL29a and RFL79 proteins. **a** Location of the predicted RFL29a and RFL79 binding sites within the *orf279* RNA, relative to the observed cleavage sites (red triangles). The predicted binding site for RFL79 encompasses nucleotides 112,975–112,956, and for RFL29a 113,148–113,129 in the *T. timopheevii* mitochondrial genome (GenBank accession: NC_022714). The key amino acids at positions 5 and 35 of each PPR motif are indicated. The colour scale reflects the strength of the match between the amino acid combination and the RNA base, calculated from in vitro binding data^43^. **b** RFL29a and RFL79 bind to *orf279* as shown by electrophoretic mobility shifts of fluorescein-labelled RNA oligonucleotides corresponding to their predicted targets. B = bound (RNA + protein) U = unbound (free RNA probe). Neither protein binds to an unrelated RNA oligonucleotide (based on *orf256* sequence). As both proteins were produced as maltose-binding protein (MBP) fusions, RNA binding with MBP alone was tested and found to be negligible. Serial protein dilutions ranging from 1.8 µM to 28.1 nM for RFL29a and RFL79, and from 0.5 µM to 7.8 nM for MBP were used for the binding assays. The final concentration of the RNA probes was 1 nM. On each gel, the left lane acts as a marker for unbound probe. More details are provided in Supplementary Fig. 7. Probe sequences are given in Supplementary Table 6. This experiment was performed three times with similar results. Source data underlying Fig. 7b are provided as a Source data file.

The RFL29a and RFL79 proteins were expressed as fusions with a polyhistidine (His) tag and maltose-binding protein (MBP) at the N-terminus (Supplementary Fig. 7). Their binding sites within the *orf279* transcript (Fig. 7) were predicted following the pattern of amino acid combinations at positions 5 and 35 within each PPR motif^42,43^. RNA oligonucleotides covering these predicted sites were synthesised and used to test the RNA-binding ability of the two proteins. Both RFL29a and RFL79 bind to their predicted RNA targets within *orf279* (Fig. 7). More details and caveats on the interpretation of these results are provided in Supplementary Fig. 7 where we show that the majority of the recombinant RFL79 and RFL29a proteins are aggregated. This is likely to negatively influence the RNA-binding ability of the proteins, and may explain why the binding to *orf279* RNA probe is weaker than observed for some other PPR proteins (apparent Kd ~ 1 µM compared with 0.1 nM for PPR10 on its favoured target^44^).

## Discussion

The potential of hybrid breeding in wheat was recognised by plant breeders at the end of the 19th century^45^. However, this technology has been under-exploited in wheat because of the difficulties in producing hybrids on an industrial scale, despite major efforts from both the commercial and academic sectors^46–48^. Of the different approaches to breeding hybrids that have been explored in wheat^49,50^, CMS, so effective in other crops, has been difficult to use because of the genetic complexity and inadequacy of fertility restoration in the hybrids^13^. An understanding of the genetic basis of CMS and fertility restoration in wheat is thus helpful for developing more effective hybrid breeding systems. We followed a positional cloning strategy coupled with a selective sequence capture approach to identify *Rf1* and *Rf3*, two of the major fertility restorer genes in wheat for T-CMS. Both genes encode members of the RFL clade of the PPR family and reside within large homeologous clusters of related genes. The wheat genome is exceptional in containing more than ~200 RFL genes^23^, compared to 20–30 in a typical plant genome, partially explaining the complexity of fertility restoration genetics in this species. *RFL79* (*Rf1*) and *RFL29a* (*Rf3*) are 89% identical in DNA sequence and 83% identical at the protein level (Supplementary Fig. 8). Despite this high sequence similarity, and the similar phenotype they are associated with, their molecular action is in fact distinct. RFL proteins are, like other PPR proteins, sequence-specific RNA-binding proteins whose specificity is governed by the amino acid residues at positions 5 and 35 of each PPR motif^42,43^. Both RFL79 and RFL29a proteins comprise 20 PPR motifs (Supplementary Fig. 9) but their comparison shows that in 13 of these motifs, the 5th/35th amino acid combinations differ between RFL79 and RFL29a. This variation is concordant with previous observations of diversifying selection acting on these residues in RFL proteins^51–53^. RFL29a and RFL79 bind to the *orf279* transcript inducing downstream cleavage of the RNA at different sites, leading in both cases to reduced accumulation of the Orf279 protein. Induced cleavage of the targeted transcript at <100 nt 3′ of the RFL-binding site appears to be a general mechanism of action of these proteins^54^. Although it is not unheard of that multiple restorer genes target the same CMS-inducing RNA (e.g. *Rf1a* and *Rf1b* in rice^55,56^), this is the first demonstration of two RFL proteins binding the same RNA and inducing its cleavage at different positions. The independent action of Rf1 and Rf3 suggests that stacking the two genes should improve fertility restoration, as indeed we observed (Fig. 3).

In most cases examined at the molecular level, CMS is correlated with aberrant, usually chimaeric, mitochondrial genes that encode small transmembrane proteins (10–35 kDa) often showing specific spatiotemporal accumulation in anthers or microspores. These proteins are thought to cause a deficiency in ATP production that triggers abnormal programmed cell death in the sporophytic tissues supporting microspore development or in the microspores themselves, ultimately leading to male sterility^14,57^. Several CMS-associated ORFs in both monocot and dicot plants contain fragments of conserved mitochondrial genes, often ones encoding subunits of the ATP synthase^14^. In many cases, only circumstantial evidence is available for the link between the presumed CMS-inducing ORF and the male-sterile phenotype, as classical genetics is not possible with uniparentally inherited organelle genes and mitochondrial transgenesis has yet to be achieved in flowering plants. In only a few cases have these links been rigorously demonstrated, namely the identification of *orf138* as the causal gene for CMS in *Brassica napus* through analysis of recombinant cybrid mitochondrial genomes^58^ and the recent identification of CMS genes in *B. napus* and rice via deliberate TALEN-induced deletions in mitochondrial DNA^59^. The previous identification of *orf256* as the probable cause of T-CMS in wheat was based on an observed correlation between *orf256* transcript processing and male fertility across only a small number of genotypes^38^, and included no near-isogenic comparisons between male-sterile and fertile lines. We show that across a larger sample of genotypes this correlation breaks down, and that neither *Rf1* nor *Rf3* influence *orf256* processing. The fact that both *RFL79* (*Rf1*) and *RFL29a* (*Rf3*) independently affect processing of the same transcript (*orf279*), and only that transcript (as judged by genome-wide RNA-Seq), is strong evidence that *orf279* is the true cause of T-CMS. The promoter and the 5′UTR region of the *orf279* gene is identical to that of the *atp8* gene. As observed for other chimaeric genes^15^, the presence of a promoter and 5′UTR region from a conserved mitochondrial gene probably explains how *orf279* is transcribed and translated by the mitochondrial gene expression machinery. CMS has been associated with different *atp8* chimaeras in sunflower, *Brassica*, and carrot^14^ including *orf138* which causes Ogura CMS and has been widely used in breeding hybrid *Brassica* crops^58,60^. Two copies of *atp8* are present in *T. aestivum* mitochondrial genomes^61^ whereas *T. timopheevii* has only one functional copy of the gene encoded in its genome (Supplementary Fig. 9). In the *T. timopheevii* mitochondrial genome, *atp8-1* is replaced by *orf279* (Supplementary Fig. 9). The highly hydrophobic nature of the additional sequence in the Orf279 extension of Atp8 is typical of other CMS-associated proteins^62,63^ and may serve to disrupt the mitochondrial inner membrane or the assembly of the ATP synthase.

The identification of *Rf1* and *Rf3* as fertility-restorer genes and *orf279* as the genetic determinant of T-CMS is an important breakthrough that can be quickly translated into applications in breeding hybrid wheat. Fertility restoration in these experiments reached 96% using transgenes with strong constitutive promoters or 93% via stacking of native *Rf1* and *Rf3* genes. Complete fertility restoration—ideally conferred by a single gene—is required to maximise the potential gains of F_1_ hybrids over conventional wheat cultivars. Ways in which that might be achieved have become clearer after this work. Higher expression of Rf1 and/or Rf3 would be expected to improve fertility, as would improving their binding to the *orf279* transcript, either of which may be achievable by rational design or mutagenesis.

Alternative routes to controlling male fertility in wheat are also worth exploring. As the expression of *orf279* in anthers is more effectively suppressed in *T. timopheevii* than in *Rf1*- or *Rf3*-containing *T. aestivum* plants, *T. timopheevii* may carry an additional, restorer gene that is yet to be identified. For example, the *Rf4* locus, located on the short arm of chromosome 6B and believed to be derived from *T. timopheevii*^64^ has yet to be cloned and characterised. Over the last few decades, the suitability of several other sterilising cytoplasms for hybrid wheat breeding has been explored including cytoplasms from several *Aegilops* species^65^ and *Hordeum chilense*^66^. The approaches we have used here to investigate the particularly complex molecular genetics of male-fertility restoration in wheat should be applicable to these other cases, too. In addition to effective fertility control systems, alterations to wheat flower architecture in parental lines to promote cross-pollination and a better understanding of the relationship between functional haplotypes and heterosis will need to be achieved for hybrid seed production to be cost-effective in wheat^11,13^. The recent explosion in the availability of genomic resources and technologies provides new avenues to overcome these limitations^23^. A viable path to the development of hybrid wheat cultivars would help to improve the stagnating seed yields in one of the world’s most important staple crops and will be critical in ensuring global food security.

## Methods

### Plant material and growth conditions

R197 and R0932E are *Rf1*-carrying restorer lines that were developed from the same initial wheat/*T. timopheevii* interspecific cross made by J. Wilson^18^. Consequently, the R197 and R0932E *Rf1* genes are identical by descent. R0934F and R0946E, Australian lines originating from a private breeding programme, and the commercial variety ‘Primepi’ carry the *Rf3* gene^32^. R0934F, R0946E and Primepi carry strong *Rf3* restorer alleles as compared to Chinese Spring (Groupe Limagrain internal evaluation). ‘Anapurna’, a maintainer line unable to restore T-CMS, was used as a negative control in this study. In addition, a *T. timopheevii* line was included as it is a fertile line expected to harbour more than one *Rf* gene able to restore T-type CMS^18^. In addition, for the purpose of this study, a Fielder maintainer line carrying T-CMS cytoplasm (Fielder*CMS) was developed. ‘Fielder’ is a standard line used in wheat transformation protocols as it is characterised by high transformation efficiency and tissue regeneration^39^. ‘Fielder’ seeds were obtained from The National Bioresource Project (NBRP)-WHEAT (Laboratory of Plant Genetics, Graduate School of Agriculture, Kyoto University, Japan). The Fielder*CMS plants are sterile when self-pollinated. Fielder*CMS and other wheat lines generated in this project are available (MTA required) upon request from Pascual Perez, Limagrain. Plants were grown in a glasshouse under standard wheat growth conditions (16 h of light period at 20 °C and 8 h of dark period at 15 °C with constant 60% humidity) until control grains of the wild type ‘Fielder’ cultivar reached maturity stage. For pollen staining and RNA-Seq analyses, anthers at three developmental stages (A, B, C) corresponding, respectively, to early (Feekes stage 10.5), mid (Feekes stage 10.5.1/10.5.2) and late anthesis (Feekes stage 10.5.3)^67^ were collected. For RNA extraction and subsequent RNA-Seq analysis, 15 anthers per sample in three biological replicates were manually collected and immediately frozen in liquid nitrogen. For pollen staining, anthers were incubated with Alexander’s stain^68^ to distinguish viable and non-viable grains and inspected under a Olympus BX51 microscope (Olympus, Tokyo, Japan) equipped with a ColorView IIIu CCD camera (Olympus, Tokyo, Japan) using analySiS getIT! v5.2 software (Olympus, Tokyo, Japan). The fertility of plants was evaluated by counting the number of seeds per spike and per spikelet and comparing with the wild type Fielder and Fielder*CMS control plants.

### Fine mapping of the genomic regions harbouring *Rf1* and *Rf3* restorer genes

We analysed three F_2_ mapping populations (R197xKalahari, R204xAlixan and R0932ExAltigo) segregating for *Rf1* and comprising 210, 218 and 212 individuals, respectively, to place the *Rf1* locus between 4.0 and 10.9 cM on chromosome 1A. The largest possible interval defined by the three mapping populations is delimited by the single-nucleotide polymorphism (SNP) markers cfn1087371 and cfn0530841 (Supplementary Table 1, Fig. 1a). Subsequent analysis of individual F_2_ recombinant plants or derived F_3_ families narrowed down the *Rf1* interval to between 7.0 and 8.9 cM and physically delimited the region by the SNP markers cfn1082074 and cfn0523990 (Supplementary Table 1, Fig. 1a). These markers were anchored to the genome sequence using the IWGSC RefSeq v1.0 reference genome^23^. Subsequently, the *Rf1* locus was fine-mapped by screening 2976 and 3072 F_3_ lines from R197xKalahari and R204xAlixan, respectively, derived from F_2_ plants heterozygous for *Rf1*. Analysis of the progenies of recombinant plants redefined the *Rf1* interval to an even shorter region between 7.5 and 8.8 cM, delimited by the cfn0522096 and cfn0527067 SNP markers (Supplementary Table 1, Fig. 1a).

To map the *Rf3* locus, three F_2_ mapping populations (TJB155xAnapurna, 2852xAltamira and AH46xR0946E) segregating for *Rf3* and comprising 217, 135 and 246 individuals, respectively, plus a doubled-haploid (DH) population (H46xR0934F) consisting of 140 individuals were phenotyped and genotyped as described above. *Rf3* was first mapped on the short arm of chromosome 1B between 18.9 and 24.2 cM on a consensus map, with the *Rf3* interval physically delimited by the SNP markers cfn0554333 and cfn0560679 (Fig. 1b). Subsequently, a joint analysis of F_3_ families genetically delimited the locus between 22.2 and 22.7 cM and physically mapped the *Rf3* interval between SNP markers cfn1249269 and BS00090770 (Fig. 1b). By using the IWGSC RefSeq v1.0 assembly the left border (cfn1249269) of the *Rf3* interval was anchored on *IWGSCWGAV02_1BS_scaffold35219* and the right border (BS00090770) was anchored on *IWGSCWGAV02_1BS_scaffold5117* (Fig. 1b, Supplementary Table 1).

### RFL capture

To design bait oligonucleotides, 33 cereal genome sequences and two transcriptome data sets were screened for open reading frames (ORFs) in six-frame translations with the *getorf* programme of the EMBOSS 6.6.0 package^69^ using parameters -minsize 279 -find 0 -reverse true. Predicted ORFs longer than 93 codons were screened for the presence of P- and PLS-class pentatricopeptide repeat (PPR) motifs using *hmmsearch* (with parameters -E 0.1 --domE 100) from the HMMER v3.1b1 package (hmmer.org) and hidden Markov models defined by *hmmbuild*^70^. HMMER output was processed using PPRfinder^71^ (https://github.com/ian-small/PPRfinder) to identify the most likely motif arrangements in each protein. Sequences containing 10 or more P-class PPR motifs were retained for further analysis, as RFL genes are generally comprised of tandem arrays of 15–20 P-class PPR motifs^51^. For identification of RFL sequences among the P-class PPRs, the OrthoMCL algorithm^72^ was used via the OrthoMCL-DB website (Release 5, 23rd July 2015) (http://www.orthomcl.org/orthomcl/) to assign P-class PPR proteins from each data set into clusters. The resulting output files were screened for groups containing 49 reference RFLs^51^. In total, 633 RFL sequences were identified in the 35 cereal data sets by OrthoMCL analysis. In addition, WGS data sets of 44 sorghum accessions including landraces and wild relatives^73^ were analysed in the same way and 517 additional RFL sequences identified. These 1199 RFL sequences underwent a pre-treatment process that included masking of sequences matching wheat organelle genome sequences (GenBank accessions NC_007579.1 and AB042240) as well as repeated elements of the wheat genome using the MIPS Repeat Elements Database v9.3^74^, the TREP Repeat Elements Database v10^75^, the TIGR Repeat Elements Database V4^76^ and RepeatMasker v.3.3.0 software (http://www.repeatmasker.org/). The remaining RFL sequences were used for capture probe design with a frequency masking algorithm intended to rule out probes that match with high copy number sequences in the targeted genome(s). A set of 62,579 DNA probes ranging from 50 to 95 nucleotides were designed. On average, probes were tiled every 36 bp on the targets. The final probes were synthetized as a probe pool. Seeds of each accession were sown and plantlets were grown in etiolated conditions. After extraction using a Nucleospin Plant II kit (Macherey-Nagel, Düren, Germany) according to the manufacturer’s recommendations, DNA samples were fragmented using a Covaris (Woburn, Massachusetts, USA) device to generate a population of fragments centred around 600 bp. Sequencing libraries were prepared with KAPA Biosystems chemistry (KAPA HTP Library Preparation Kit Illumina 96 Rxn; Roche, Basel, Switzerland) according to the manufacturer’s recommendations. The libraries were then specifically enriched in RFL sequences using the probe pool (SeqCap EZ Developer Library, 12 Reactions; Roche) and two consecutive rounds of sequence capture, referred to as the double capture protocol, as recommended by the manufacturer in case of limited cumulated size of the targets in the genome. The efficiency of the capture was confirmed via the measurement of targeted sequence enrichment and untargeted sequence depletion (chloroplast genome sequence) using qPCR and primer sequences given in Supplementary Table 6. Ultimately libraries were pooled and sequenced in paired-end mode with 300 nt read length on an Illumina MiSeq platform (Illumina, San Diego, California, USA). Overlapping paired reads were merged into a single sequence (to maximise read length) using *bbmerge* from the *bbmap* package v.35.x (https://sourceforge.net/projects/bbmap/) with the parameters qtrim2 = t trimq = 10,15,20 minq = 12 mininsert = 150. Read pairs that could not be merged were discarded. The merged reads were downsampled to 300,000 reads using *reformat.sh* in the *bbmap* package (samplereadstarget = 300000). The merged and downsampled reads were assembled with the de novo assembler included in Geneious 8 (set to Medium Sensitivity/Fast) (http://www.geneious.com/). Finally, contigs composed of more than 100 merged reads were retained for further analysis, with most of these composed of over 1000 reads. Sequences encoding RFL proteins were identified within these contigs as described at the beginning of this section. Finally, to identify putatively orthologous RFL sequences across all eight accessions and *T. timopheevii*, the 2022 RFL ORFs (Supplementary Table 4) were clustered using CD-HIT^36^ (settings -c 0.96 -n 5 -G 0 -d 0 -AS 60 -A 105 -g 1).

### Cloning of candidate genes and *Agrobacterium*-mediated transformation

*RFL79* derived from R197 (sequence: R197.300k_Assembly_Contig_120_1), *RFL104* sequence derived from R0932E (sequence: R0932E.300k_Assembly_Contig_82_1), *RFL29a* sequence derived from R0934F (sequence: R0934F.300k_Assembly_Contig_78_1), *RFL29b* derived from Chinese Spring (sequence: TraesCS1B02G038500.1) were cloned via a Golden Gate reaction into the destination binary plasmid pBIOS10746. The coding sequences of the candidate genes were cloned between the strong *Zea mays* ubiquitin promoter (*ZmUbi*) sequence containing the first intron and the *SbHSP* terminator (3′-UTR) from the *Sorghum bicolor Sb03g006880* gene encoding a heat shock protein (HSP18.2). The screening cassette consists of the *T. aestivum* HMWG promoter, *ZsGreen* (green fluorescent protein derived from *Zoanthus*) and *tNos* terminator sequence. The selection cassette consists of a rice actin gene promoter and first intron, the *bialaphos resistance* (*Bar*) gene and *tNos* terminator sequence. DNA fragments corresponding to the screening cassette and to the selection cassette were purchased from GenScript (Piscataway, New Jersey, USA) and used to create destination vectors. In parallel, the coding sequences of the candidates were cloned via restriction enzyme reaction, between the native *T. aestivum* promoter and 3′-UTRs into the destination binary plasmid pBIOS10747. The promoter sequences were obtained either from available BAC clones (*RFL29a*), from the IWGSC RefSeq v1.0 sequence (*RFL29b*) or generated by using several rounds of sequence capture protocol^77^. The corresponding sequences were synthesized by GenScript. For the *RFL29a* + *RFL79* double construct, the coding sequences of *RFL29a* and *RFL79* were cloned under the control of their endogenous promoters and terminators into the pBIOS10747 plasmid. The binary destination vectors pBIOS10746 and pBIOS10747 are a derivative of the binary vector pMRT^78^. All binary plasmids described above were transformed into *Agrobacterium* EHA105. Fielder*CMS as well as conventional Fielder cultivar were transformed with these *Agrobacterium* strains as described by ref. ^39^. Transgenic events were generated for each of the constructs. For the *ZmMac2* experiments, the *ZmMac2::Barnase* cassette was cloned as a *Kpn*I fragment from the *ZmMac2::Barnase* vector^40^ into the *Kpn*I site of an pSB11-based plant binary transformation vector (pGB53)^79^. This construct was used to transform NB1 wheat^80^. The *ZmMac2* sequence was synthesized with suitable restriction sites by GenScript and then cloned with the *RFL29a* coding sequence and the *SbHSP* 3′-UTR via a Golden Gate reaction into a derivative of the binary plasmid pBIOS10746. The corresponding T-DNA is described in Fig. 4a. The *SbHSP* 3′-UTR was replaced by that of *RFL29a* by restriction cloning (exchange of a *Sna*BI*-Asc*I fragment) to create the *ZmMac2::RFL29a::RFL29a* cassette (Fig. 4a). These binary plasmids were then transferred into *Agrobacterium* EHA105 and used to transform Fielder*CMS as described above.

### Genotyping of *Rf1* and *Rf3* transformants

For each plant, genomic DNA was isolated using 50 mg of fresh leaf material with a microextraction method with the DNeasy 96 Plant kit (Qiagen, Hilden, Germany) following the manufacturer’s instructions. Real-time PCR was carried out in 384-well reaction plates to estimate the number of transgenes integrated per plant. Reactions were multiplexed to simultaneously amplify the Selection Cassette Bar transgene and the endogenous gene GaMyb (GenBank accession numbers EF114937.1, EF114922.1, EF114913.1). For each sample, the reaction mixture contained 5 μL 2x TaqMan Genotyping Master mix (Applied Biosystems, Foster City, California, USA), 500 nM of each primer, 200 nM of each probe and 2 μL of genomic DNA (5–10 ng) in a final volume of 10 μL. Two replicates were performed for each sample and a calibrator sample (known 1 copy of Bar control). The PCR was run on a 7900HT Real-Time System (Applied Biosystems) using the following thermal cycling conditions: 2 min at 50 °C, 10 min at 95 °C and 40 cycles with 15 s at 95 °C and 1 min at 60 °C. Results were analysed with SDS Software v2.4 (Applied Biosystems) to estimate transgene copy number applying the 2^−ΔΔCT^ method^81^. Primers and probes were designed using the Primer Express software v2.0 (Applied Biosystems) and are listed in Supplementary Table 6. An expression value of ~1 indicates single insertion of the transgene whereas a value higher than 1 indicates multiple insertions.

### RNA analyses

RNA was extracted from plants with an RNeasy Plant Mini Kit (Qiagen) according to the manufacturer’s instructions and treated with TURBO™ DNase (Invitrogen, Carlsbad, California, USA). For *orf256* and *orf279* expression analyses, cDNA was synthesized using Superscript III (Invitrogen) and random primers (Invitrogen). The amplification was performed with the gene-specific primers listed in Supplementary Table 6. Northern blotting was performed as previously described^82^. In short, 5–10 μg of total RNA per sample were separated on a 1.2% denaturing agarose gel and transferred onto Amersham Hybond N + membrane (Cytiva, formerly GE Healthcare Life Sciences, Marlborough, Massachusetts, USA). DNA fragments corresponding to a part of the coding sequence of *orf256* were amplified from cDNA of *T. timopheevii* using primers listed in Supplementary Table 6 and cloned into pGEM‐T Easy vector (Promega, Madison, Wisconsin, USA). Positive clones served as a template for in vitro transcription using Biotin-14-CTP (Invitrogen) following the protocol supplied with the MAXIscript® In Vitro Transcription Kit (Invitrogen). Pre‐hybridization of the membrane was carried out at 65 °C for 1 h in PerfectHyb Plus Hybridization Buffer (MilliporeSigma, formerly known as Sigma-Aldrich, Burlington, Massachusetts, USA). Hybridization with RNA probes was carried out overnight at 65 °C in a fresh aliquot of the hybridisation buffer. Three 15‐min washes at 37 °C in 2× saline-sodium citrate (SSC)/0.1% sodium dodecyl sulfate (SDS), 1×SSC/0.1% SDS and 0.5×SSC/0.1% SDS, respectively, were performed. The signal of the probes was detected using the Thermo Scientific Pierce Chemiluminescent Nucleic Acid Detection Module Kit (Thermo Fisher Scientific, Waltham, Massachusetts, USA) and recorded using GE ImageQuant‐RT ECL Imager (Cytiva). One microgram of total RNA was used for cDNA synthesis and amplification of 5′ ends using the SMARTer RACE 5′/3′ Kit (Takara Bio, Kusatsu, Shiga, Japan) following the manufacturer’s instructions. PCR products were gel-eluted, cloned into pGEM‐T Easy vector (Promega) and sequenced at Macrogen (Seoul, South Korea). Gene-specific primer sequences (GSPs) are given in Supplementary Table 6.

RNA-Seq libraries were made with the TruSeq Stranded Total RNA with Ribo-Zero Plant Prep Kit (Illumina) and sequenced on a HiSeq 4000 or NovaSeq 6000 platform (Illumina) with 100 or 150 nt paired-end reads at Novogene (Novogene, China). Reads were adapter-trimmed with *bbduk* (parameters ktrim = r k = 23 mink = 11 hdist = 1 tpe tbo ftm = 5). Salmon (v1.3.0)^83^ was used to assign reads to transcripts and calculate transcripts per million values. For nuclear/cytosolic transcripts, the IWGSC 1.1 annotations were used as a reference, but with the Chinese Spring RFL transcripts replaced by captured RFL sequences from the sequenced genotype. For mitochondrial transcripts, annotated coding sequences from the *T. timopheevii* mitochondrial genome (NC_022714) were used, supplemented with ten *T. timopheevii*-specific ORFs of over 100 codons. Where relative gene expression was evaluated (Fig. 2f), read counts were normalised across samples using the pseudoreference approach of DeSeq2^84^. For analysis of read coverage, adapter-trimmed reads were mapped to the *T. timopheevii* mitochondrial genome (NC_022714) with *bbmap*^85^. Multi-mapped reads were distributed randomly between the best-matching sites and rRNA regions were masked (because rRNA depletion was inconsistent across samples). Regions identical to plastid DNA were masked to avoid cross-mapped plastid reads. Read coverage was normalised by dividing by mean coverage depth excluding the masked regions.

### Protein extraction and immunological analysis

Mitochondria were enriched from 11-day-old wheat seedlings grown on vermiculite in the dark according to a previously published protocol^86^. Wheat seedlings were cut into 0.5–1 cm pieces and ground in pre-cooled mortar and pestle in grinding medium (0.3 M sucrose, 25 mM tetrasodium pyrophosphate, 2 mM ethylenediaminetetraacetic acid (EDTA), 10 mM KH_2_PO_4_ and 1% polyvinylpyrrolidone-40, 1% bovine serum albumin (BSA), 10 mM ascorbic acid, pH 7.5 with H_3_PO_4_) with addition of acid-washed sand in a cold room. The suspension was then filtered through 4 layers of Miracloth (MilliporeSigma formerly known as Millipore). The homogenate was centrifuged for 5 min at 1000 × *g* at 4 °C in an Avanti J-26XP centrifuge (Beckman Coulter, Brea, California, USA). The pellet was discarded and the supernatant spun down again for 20 min at 21,000 × *g* at 4 °C. The resulting pellet was resuspended in residual supernatant using a small paintbrush and 500 μL of 1× wash buffer (0.3 M sucrose, 10 mM TES, 0.1% BSA, pH 7.5) and layered over a 10 mL 18%/24%/50% Percoll step gradient. Percoll gradient solutions were prepared by mixing appropriate amounts of Percoll solution (Cytiva), 2× wash buffer (0.6 M sucrose, 20 mM TES, 0.2% BSA, pH 7.5) and sterile water. The tubes were balanced and centrifuged at 40,000 × *g* for 45 min at 4 °C with brakes off. Intact mitochondria that formed a light yellow/whitish band towards the bottom of the tube were washed twice in 1× wash buffer and centrifuged for 20 min at 21,000 × *g* at 4 °C. When the available plant material was scarce, the Percoll gradient purification step was omitted and pellets after the second centrifugation step were washed in 1× wash buffer and used directly for further analyses. After the washing step, the mitochondrial pellets were resuspended in ACA buffer (0.75 M aminocaproic acid, 0.5 mM EDTA, 50 mM Bis-Tris pH 7.0) and treated with 1% *n*-dodecyl-β-d-maltoside solution to release membrane-bound proteins for 20 min on ice. After a 10 min-centrifugation step at 20,800 × *g* the supernatant was transferred into a new tube and mixed with 4 volumes of acetone supplemented with 1% β-mercaptoethanol to precipitate the proteins. After 10 min centrifugation step at 20,800 × *g* the proteins were resuspended directly in protein loading buffer (1 M Tris-HCl pH 6.8, 2% SDS, 10% glycerol, 0.0006% bromophenol blue, 5% β-mercaptoethanol), heated at 95 °C for 10 min and separated on a 15% Stain-Free™ SDS-PAGE gel (Bio-Rad, Hercules, California, USA). The transfer onto Amersham PVDF blotting membrane (Cytiva) was performed using a semi-dry blot apparatus (Bio-Rad). The antibody against recombinant Orf279 protein was custom-made at GenScript. Part of the unique region of Orf279 encompassing amino acid residues 137–279 was overexpressed in *E. coli* and used for immunisation of two rabbits. For immunological detection the anti-Orf279 antibody was diluted 1:500 and the anti‐rabbit antibodies conjugated to horseradish peroxidase (MilliporeSigma) were diluted 1:10,000 in TBST (Tris-buffered saline, 0.1% Tween 20) buffer. The chemiluminescent signals were detected with Clarity Western ECL Substrate (Bio-Rad) in an ImageQuant-RT ECL Imager (Cytiva).

### RNA electrophoretic mobility shift assay

Recombinant RFL79 and RFL29a proteins were expressed as maltose-binding protein-fusions and purified by affinity chromatography as described previously for other PPR proteins^87^. The coding sequences of *RL79* and *RFL29a* were amplified from genomic DNA using the PrimeStar HS DNA polymerase (Takara Bio) with primers containing *attB* sites for Gateway cloning technology (Invitrogen) (Supplementary Table 6). The obtained PCR products were subcloned into pDONR207 vector (Invitrogen) with Gateway BP Clonase II enzyme mix (Invitrogen). The positive clones were identified by sequencing at Macrogen and cloned into the expression vector pETG-41K (EMBL, Heidelberg, Germany) with Gateway LR Clonase II enzyme mix (Invitrogen). The pETG-41K vector allows an addition of 6xhistidine (His) tag that can be used for Ni-NTA purification and an MBP (maltose-binding protein) tag at the N-terminus of the recombinant protein. For protein expression, the chemically competent cells of *E. coli* C41(DE3) strain (MilliporeSigma) were used. Transformed cells were grown in 500 mL LB (1× Luria-Bertani and 50 mM Tris-HCl, pH 8.0) medium at 37 °C and 220 rpm until the OD_600_ reached 0.4. Glass flasks with the cultures were then transferred on ice for 15 min and protein expression was initiated by addition of isopropyl β-d-1-thiogalactopyranoside (Promega) to a final concentration of 0.1 mM. The cultures were grown at 16 °C and 220 rpm overnight and harvested by centrifugation at 3000 × *g* for 15 min in an Avanti J-26XP centrifuge (Beckman Coulter). Bacterial pellets were dissolved in 35 mL of lysis buffer (0.5 M NaCl, 50 mM HEPES-KOH pH 8, 10 mM imidazole, and 7 mM β-mercaptoethanol) and cells were disrupted by homogenization with Avestin Emulsiflex C5 (Avestin, Ottawa, Ontario, Canada). Soluble protein fractions were cleared from cell debris by centrifugation for 15 min at 13,000 × *g* at 4 °C, incubated with Profinity IMAC Ni-charged resin (Bio-Rad) on a rotating wheel for 1 h and packed into empty Econo-Pac gravity columns (Bio-Rad). After three washes with 1x wash buffer (0.5 M NaCl, 50 mM HEPES-KOH pH 8, 7 mM β-mercaptoethanol, 20 mM imidazole) proteins were eluted from the Ni-charged resin with elution buffer (0.5 M NaCl, 50 mM Tris-HCl, pH 8, 250 mM imidazole) and dialysed overnight at 4 °C in dialysis buffer (0.5 M NaCl, 50 mM Tris-HCl, pH 8, 50% glycerol, 2 mM EDTA, 7 mM β-mercaptoethanol) with slow stirring on a magnetic mixer. The concentrations of the dialysed proteins were measured at ND-1000 NanoDrop spectrophotometer (Thermo Fisher Scientific). More details on protein purification and analysis are given in Supplementary Fig. 7. The sequences of fluorescein-labelled oligonucleotides used in the REMSAs performed following the previously published protocol^87^ are listed in Supplementary Table 6. Briefly, 10 μL of binding buffer consisting of 1× THE (34 mM Tris, 66 mM HEPES, 0.1 mM EDTA pH 8), 0.2 M NaCl, 5 mM dithiothreitol, 5 mg/mL heparin, 0.1 mg/mL BSA, and 8 units of RNaseOUT (Invitrogen) were mixed with 5 μL of dialysed-protein dilution and incubated at room temperature for 10 min. The 5′-fluorescein-labelled probes (MilliporeSigma) were heated for 2 min at 94 °C and incubated on ice for at least 4 min. 10 μL of denatured probes were added to the 15 μL binding reaction for a total reaction volume of 25 μL and incubated at 25 °C for 15 min. 15 μL of the binding reaction were loaded onto a pre-run 5% native polyacrylamide gel using the 1× THE as a running buffer in a cold room. After the run gels were imaged with a Typhoon Biomolecular Imager (Cytiva). Fluorescein-labelled probes were excited by a 488 nm laser and detected through a 520 nm band-pass emission filter.

### Reporting summary

Further information on experimental design is available in the Nature Research Reporting Summary linked to this paper.

## Supplementary information

Supplementary Information

Peer Review File

Reporting Summary

Description of Additional Supplementary Files

Supplementary Dataset 1-2

## Data Availability

Data supporting the findings of this work are available within the paper and its supplementary information files. A reporting summary for this article is available as a supplementary information file. The datasets and materials generated and analysed during the current study are available from the corresponding author upon request. The sequencing data from this study is available from the National Centre for Biotechnology Information Sequence Read Archive under the BioProject accession PRJNA595448 for the sequence capture data, and accessions PRJNA595431 and PRJNA675907 for the RNA-Seq data. Assembled sequence capture data is available from GenBank with the accession codes MT014021-MT015390. Source data are provided with this paper.

## Source data

Source Data

## Code availability

RNA-Seq counts and Python code used for plotting Figs. 2d–f, 3c and 5 and Supplementary Figures 2 and 5 are available from Dryad [https://datadryad.org/stash/dataset/doi:10.5061/dryad.6djh9w10d]^88^.

## References

[1] Godfray HCJ (2010). Food security: the challenge of feeding 9 billion people. Science.

[2] Tilman D, Cassman KG, Matson PA, Naylor R, Polasky S (2002). Agricultural sustainability and intensive production practices. Nature.

[3] Foley JA (2011). Solutions for a cultivated planet. Nature.

[4] Bevan MW (2017). Genomic innovation for crop improvement. Nature.

[5] Hickey LT (2019). Breeding crops to feed 10 billion. Nat. Biotechnol..

[6] Tester M, Langridge P (2010). Breeding technologies to increase crop production in a changing world. Science.

[7] Langridge, P. *Achieving Sustainable Cultivation of Wheat. Volume 2: Cultivation Techniques* (Taylor & Francis, 2017).

[8] Ray DK, Mueller ND, West PC, Foley JA (2013). Yield trends are insufficient to double global crop production by 2050. PLoS ONE.

[9] Grassini P, Eskridge KM, Cassman KG (2013). Distinguishing between yield advances and yield plateaus in historical crop production trends. Nat. Commun..

[10] Longin CFH (2013). Hybrid wheat: quantitative genetic parameters and consequences for the design of breeding programs. Theor. Appl. Genet..

[11] Gupta PK (2019). Hybrid wheat: past, present and future. Theor. Appl. Genet..

[12] Mühleisen J, Piepho H-P, Maurer HP, Longin CFH, Reif JC (2014). Yield stability of hybrids versus lines in wheat, barley, and triticale. Theor. Appl. Genet..

[13] Whitford R (2013). Hybrid breeding in wheat: technologies to improve hybrid wheat seed production. J. Exp. Bot..

[14] Chen L, Liu Y-G (2014). Male sterility and fertility restoration in crops. Annu. Rev. Plant Biol..

[15] Chase CD (2007). Cytoplasmic male sterility: a window to the world of plant mitochondrial-nuclear interactions. Trends Genet..

[16] Bohra A, Jha UC, Adhimoolam P, Bisht D, Singh NP (2016). Cytoplasmic male sterility (CMS) in hybrid breeding in field crops. Plant Cell Rep..

[17] Luo D (2013). A detrimental mitochondrial-nuclear interaction causes cytoplasmic male sterility in rice. Nat. Genet..

[18] Wilson AJ (1962). Male sterility interaction of the *Triticum aestivum* nucleus and *Triticum timopheevi* cytoplasm. Wheat Int. Serv..

[19] Kazama T, Nakamura T, Watanabe M, Sugita M, Toriyama K (2008). Suppression mechanism of mitochondrial ORF79 accumulation by Rf1 protein in BT-type cytoplasmic male sterile rice. Plant J..

[20] Bentolila S, Alfonso AA, Hanson MR (2002). A pentatricopeptide repeat-containing gene restores fertility to cytoplasmic male-sterile plants. Proc. Natl Acad. Sci. USA.

[21] Kim Y-J, Zhang D (2018). Molecular control of male fertility for crop hybrid breeding. Trends Plant Sci..

[22] Kotchoni SO, Jimenez-Lopez JC, Gachomo EW, Seufferheld MJ (2010). A new and unified nomenclature for male fertility restorer (RF) proteins in higher plants. PLoS ONE.

[23] International Wheat Genome Sequencing Consortium (IWGSC (2018). Shifting the limits in wheat research and breeding using a fully annotated reference genome. Science.

[24] Bahl PN, Maan SS (1973). Chromosomal location of male fertility restoring genes in six lines of common wheat. Crop Sci..

[25] Maan SS (1985). Genetic analyses of male-fertility restoration in wheat: isolation, penetrance, and expressivity of Rf genes. Crop Sci..

[26] Geyer M, Albrecht T, Hartl L, Mohler V (2018). Exploring the genetics of fertility restoration controlled by *Rf1* in common wheat (*Triticum aestivum* L.) using high-density linkage maps. Mol. Genet. Genomics.

[27] Du H, Maan SS, Hammond JJ (1991). Genetic analyses of male-fertility restoration in wheat: effects of aneuploidy. Crop Sci..

[28] Kojima T, Tsujimoto H, Ogihara Y (1997). High-resolution RFLP mapping of the fertility restoration (*Rf3*) gene against *Triticum timopheevi* cytoplasm located on chromosome 1BS of common wheat. Genes Genet. Syst..

[29] Ahmed TA, Tsujimoto H, Sasakuma T (2001). QTL analysis of fertility-restoration against cytoplasmic male sterility in wheat. Genes Genet. Syst..

[30] Geyer M, Bund A, Albrecht T, Hartl L, Mohler V (2016). Distribution of the fertility-restoring gene *Rf3* in common and spelt wheat determined by an informative SNP marker. Mol. Breed..

[31] Würschum T, Leiser WL, Weissmann S, Maurer HP (2017). Genetic architecture of male fertility restoration of *Triticum timopheevii* cytoplasm and fine-mapping of the major restorer locus *Rf3* on chromosome 1B. Theor. Appl. Genet..

[32] Ma Z-Q, Sorrells ME (1995). Genetic analysis of fertility restoration in wheat using restriction fragment length polymorphisms. Crop Sci..

[33] Tahir CM, Tsunewaki K (1969). Monosomic analysis of *Triticum spelta* var. *duhamelianum*, a fertility-restorer for *T. timopheevi* cytoplasm. Jpn. J. Genet..

[34] Emanuelsson O, Brunak S, von Heijne G, Nielsen H (2007). Locating proteins in the cell using TargetP, SignalP and related tools. Nat. Protoc..

[35] Kučera L (1982). Monosomic analysis of fertility restoration in common wheat ‘Prof. Marchal’. Euphytica.

[36] Li W, Godzik A (2006). Cd-hit: a fast program for clustering and comparing large sets of protein or nucleotide sequences. Bioinformatics.

[37] Song J, Hedgcoth C (1994). A chimeric gene (*orf256*) is expressed as protein only in cytoplasmic male-sterile lines of wheat. Plant Mol. Biol..

[38] Song J, Hedgcoth C (1994). Influence of nuclear background on transcription of a chimeric gene (*orf256*) and *coxI* in fertile and cytoplasmic male sterile wheats. Genome.

[39] Ishida Y, Tsunashima M, Hiei Y, Komari T (2015). Wheat (*Triticum aestivum* L.) transformation using immature embryos. Methods Mol. Biol..

[40] Paul, W., Scott, R. J., Hird, D. & Hodge, R. Tapetum-specific promoters. *US Patent* (2006).

[41] Krogh A, Larsson B, von Heijne G, Sonnhammer EL (2001). Predicting transmembrane protein topology with a hidden Markov model: application to complete genomes. J. Mol. Biol..

[42] Barkan A (2012). A combinatorial amino acid code for RNA recognition by pentatricopeptide repeat proteins. PLoS Genet..

[43] Yan J (2019). Delineation of pentatricopeptide repeat codes for target RNA prediction. Nucleic Acids Res..

[44] Prikryl J, Rojas M, Schuster G, Barkan A (2011). Mechanism of RNA stabilization and translational activation by a pentatricopeptide repeat protein. Proc. Natl Acad. Sci. USA.

[45] E. H (1886). ‘Hybrid’ wheat. Nature.

[46] Miedaner T, Schulthess AW, Gowda M, Reif JC, Longin CFH (2017). High accuracy of predicting hybrid performance of *Fusarium* head blight resistance by mid-parent values in wheat. Theor. Appl. Genet..

[47] Thorwarth P (2018). Higher grain yield and higher grain protein deviation underline the potential of hybrid wheat for a sustainable agriculture. Plant Breed..

[48] Würschum T (2018). Exploiting the *Rht* portfolio for hybrid wheat breeding. Theor. Appl. Genet..

[49] Athwal DS, Phul PS, Minocha JL (1967). Genetic male sterility in wheat. Euphytica.

[50] Tucker EJ (2017). Molecular identification of the wheat male fertility gene *Ms1* and its prospects for hybrid breeding. Nat. Commun..

[51] Fujii S, Bond CS, Small ID (2011). Selection patterns on restorer-like genes reveal a conflict between nuclear and mitochondrial genomes throughout angiosperm evolution. Proc. Natl Acad. Sci. USA.

[52] Melonek J, Stone JD, Small I (2016). Evolutionary plasticity of restorer-of-fertility-like proteins in rice. Sci. Rep..

[53] Melonek J (2019). High intraspecific diversity of *Restorer-of-fertility-like* genes in barley. Plant J..

[54] Colas des Francs-Small C, Vincis Pereira Sanglard L, Small I (2018). Targeted cleavage of *nad6* mRNA induced by a modified pentatricopeptide repeat protein in plant mitochondria. Commun. Biol..

[55] Wang Z (2006). Cytoplasmic male sterility of rice with boro II cytoplasm is caused by a cytotoxic peptide and is restored by two related PPR motif genes via distinct modes of mRNA silencing. Plant Cell.

[56] Hu J (2012). The rice pentatricopeptide repeat protein RF5 restores fertility in Hong-Lian cytoplasmic male-sterile lines via a complex with the glycine-rich protein GRP162. Plant Cell.

[57] Touzet P, Meyer EH (2014). Cytoplasmic male sterility and mitochondrial metabolism in plants. Mitochondrion.

[58] Bonhomme S (1992). Sequence and transcript analysis of the Nco2.5 Ogura-specific fragment correlated with cytoplasmic male sterility in *Brassica* cybrids. Mol. Gen. Genet..

[59] Kazama T (2019). Curing cytoplasmic male sterility via TALEN-mediated mitochondrial genome editing. Nat. Plants.

[60] Yamagishi H, Bhat SR (2014). Cytoplasmic male sterility in Brassicaceae crops. Breed. Sci..

[61] Ogihara Y (2005). Structural dynamics of cereal mitochondrial genomes as revealed by complete nucleotide sequencing of the wheat mitochondrial genome. Nucleic Acids Res..

[62] Duroc Y (2005). Biochemical and functional characterization of ORF138, a mitochondrial protein responsible for Ogura cytoplasmic male sterility in Brassiceae. Biochimie.

[63] Dewey RE, Timothy DH, Levings CS (1987). A mitochondrial protein associated with cytoplasmic male sterility in the T cytoplasm of maize. Proc. Natl Acad. Sci. USA.

[64] Li Z (2014). SSR analysis and identification of fertility restorer genes *Rf1* and *Rf4* of *Triticum timopheevii* cytoplasmic male sterility (T-CMS) in wheat (Triticum aestivum L.). Non. Ye Sheng Wu Ji Shu Xue Bao.

[65] Mukai Y, Tsunewaki K (1979). Basic studies on hybrid wheat breeding. Theor. Appl. Genet..

[66] Martín AC, Atienza SG, Ramírez MC, Barro F, Martín A (2008). Male fertility restoration of wheat in *Hordeum chilense* cytoplasm is associated with 6HchS chromosome addition. Aust. J. Agric. Res..

[67] Large, E. C. Growth stages in cereals. Illustration of the Feekes scale. *Plant Pathol*. **3**, 128–129 (1954).

[68] Alexander MP (1969). Differential staining of aborted and nonaborted pollen. Stain Technol..

[69] Rice P, Longden I, Bleasby A (2000). EMBOSS: the European molecular biology open software suite. Trends Genet..

[70] Cheng S (2016). Redefining the structural motifs that determine RNA binding and RNA editing by pentatricopeptide repeat proteins in land plants. Plant J..

[71] Gutmann B (2020). The expansion and diversification of pentatricopeptide repeat RNA-editing factors in plants. Mol. Plant.

[72] Li L, Stoeckert CJ, Roos DS (2003). OrthoMCL: identification of ortholog groups for eukaryotic genomes. Genome Res..

[73] Mace ES (2013). Whole-genome sequencing reveals untapped genetic potential in Africa’s indigenous cereal crop sorghum. Nat. Commun..

[74] Nussbaumer T (2013). MIPS PlantsDB: a database framework for comparative plant genome research. Nucleic Acids Res..

[75] Wicker, T., Matthews, D. E. & Keller, B. TREP: a database for Triticeae repetitive elements. *Trend. Plant Sci.* **7**, 561–562 (2002).

[76] Ouyang S, Buell CR (2004). The TIGR Plant Repeat Databases: a collective resource for the identification of repetitive sequences in plants. Nucleic Acids Res..

[77] Inagaki S, Henry IM, Lieberman MC, Comai L (2015). High-throughput analysis of T-DNA location and structure using sequence capture. PLoS ONE.

[78] Gruber, V. & Comeau, D. Synthetic vectors, transgenic plants containing them, and methods for obtaining them. *WO2001018192A2* (2001).

[79] Ishida Y (1996). High efficiency transformation of maize (*Zea mays* L.) mediated by *Agrobacterium tumefaciens*. Nat. Biotechnol..

[80] Risacher T, Craze M, Bowden S, Paul W, Barsby T (2009). Highly efficient *Agrobacterium*-mediated transformation of wheat via in planta inoculation. Methods Mol. Biol..

[81] Livak KJ, Schmittgen TD (2001). Analysis of relative gene expression data using real-time quantitative PCR and the 2^−ΔΔCT^ Method. Methods.

[82] Chateigner-Boutin A-L (2011). OTP70 is a pentatricopeptide repeat protein of the E subgroup involved in splicing of the plastid transcript. rpoC1. Plant J..

[83] Patro R, Duggal G, Love MI, Irizarry RA, Kingsford C (2017). Salmon provides fast and bias-aware quantification of transcript expression. Nat. Methods.

[84] Love MI, Huber W, Anders S (2014). Moderated estimation of fold change and dispersion for RNA-seq data with DESeq2. Genome Biol..

[85] Bushnell, B. *BBMap Short-read Aligner, And Other Bioinformatics Tools* (2016).

[86] Huang S, Jacoby RP, Millar AH, Taylor NL (2014). Plant mitochondrial proteomics. Methods Mol. Biol..

[87] Kindgren P, Yap A, Bond CS, Small I (2015). Predictable alteration of sequence recognition by RNA editing factors from *Arabidopsis*. Plant Cell.

[88] Small, I. et al. Data from: the genetic basis of cytoplasmic male sterility and fertility restoration in wheat. 10.5061/dryad.6djh9w10d (2021). 10.1038/s41467-021-21225-0 PMC7884431 33589621

