# Supplementary material for: The genetic basis of cytoplasmic male sterility and fertility restoration in wheat
Source: Nat Commun. 2021 Feb 15;12:1036. doi: 10.1038/s41467-021-21225-0 (PMC7884431; doi:10.1038/s41467-021-21225-0)
Supplement: Supplementary file 1 — Supplementary Information [file 41467_2021_21225_MOESM1_ESM.pdf]

The genetic basis of cytoplasmic male sterility and fertility restoration in wheat

Melonek *et al.*

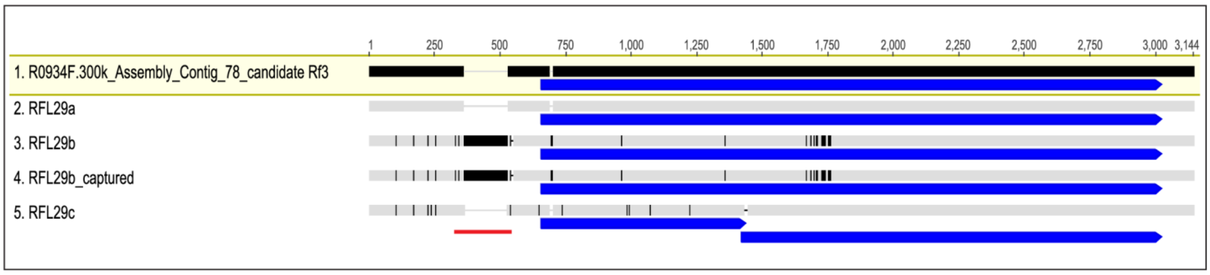

Supplementary Figure 1. Sequence alignment. Analysis of sequences present in RFL group 29. In this visualization, each vertical bar in the aligned sequences represents a mismatch compared to R0934F.300k_Assembly_Contig_78 sequence that was selected as *Rf3* candidate. An insertion in the putative 5' UTR of *RFL29b* is underlined in red and the open reading frames are shown in blue. Source data are provided as a Source Data file.

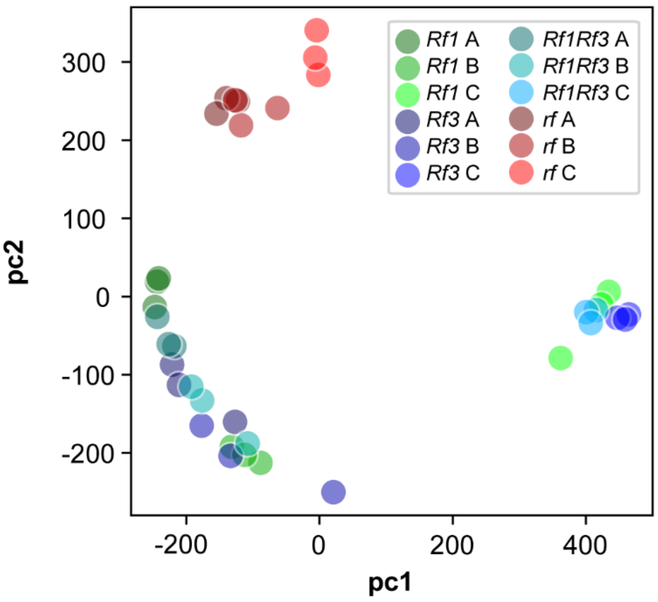

Supplementary Figure 2. Principal components analysis (PCA) of the read counts for each transcript from anthers collected at three developmental stages: early heading (A), early-mid anthesis (B) and late anthesis (C) in the fertile and sterile plants. n=3. The first principal component (pc1, 39% of variance) distinguishes the differentiation between the three stages in the fertile genotypes and the second principal component (pc2, 17% of variance) distinguishes strong differentiation between the fertile and sterile genotypes. *Rf1* corresponds to R0932E, *Rf3* to R0946E, *Rf1Rf3* to R0934F and *rf* to Fielder*CMS line. RNA-Seq counts and Python code used for plotting the figure are available from Dryad (https://doi.org/10.5061/dryad.6djh9w10d).

a

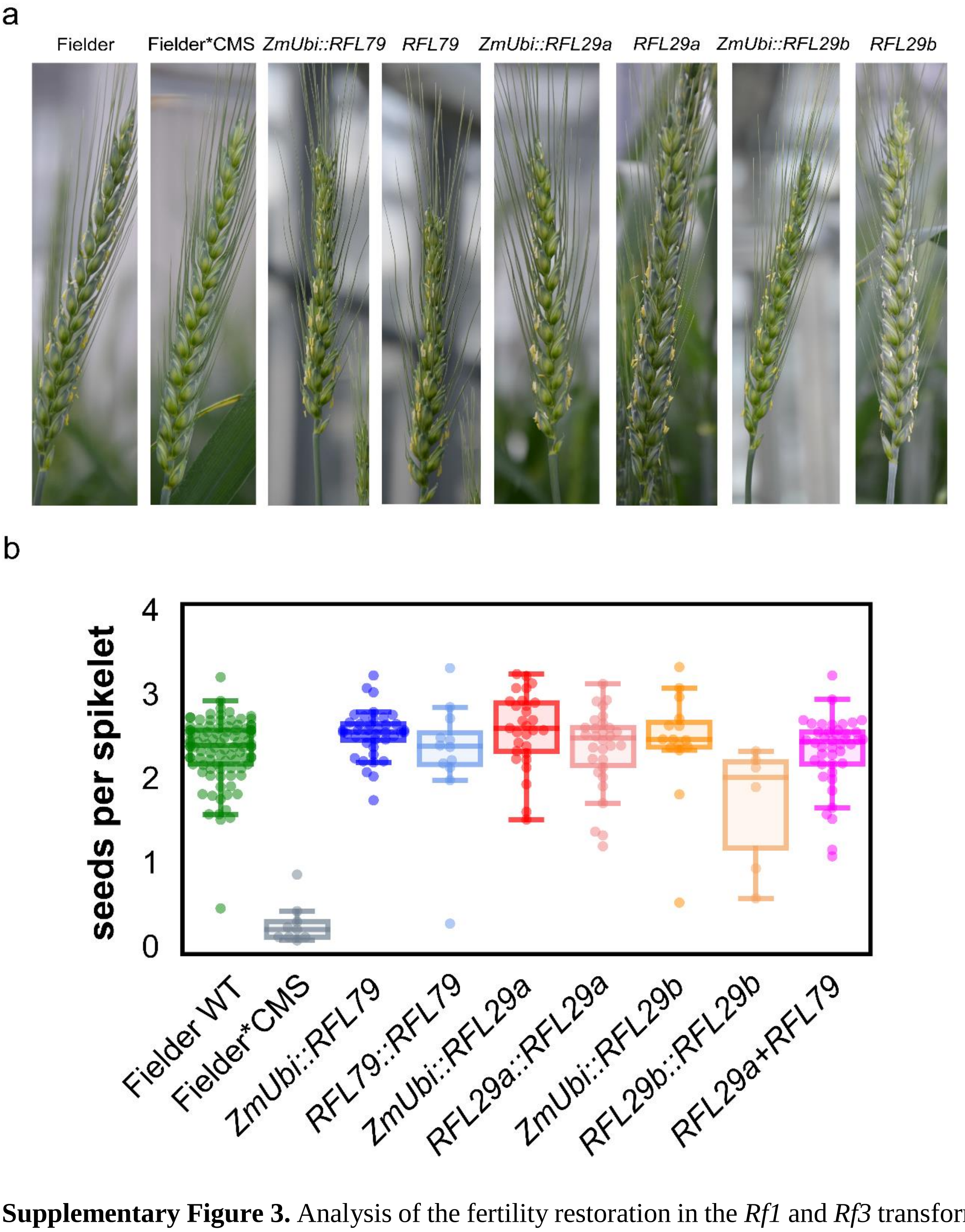

Supplementary Figure 3. Analysis of the fertility restoration in the *Rf1* and *Rf3* transformants compared to Fielder*CMS line. (**a**) Examples of the flowering phenotype of the transgenic plants. (**b**) Fertility restoration scores. The fertility scores were calculated by dividing the total number of seeds threshed from a spike by the number of counted spikelets. Centre line, median; box limits, upper and lower quartiles; whiskers, 1.5x interquartile range. Number of spikelets analyzed per line: Fielder WT n=20777, Fielder*CMS n=2386, *ZmUbiRFL79* n=5930, *RFL79* n=1660, *ZmUbiRFL29a* n=3819, *RFL29a* n=3633, *ZmUbiRFL29b* n=1934, *RFL29b* n=809, *RFL29a+RFL79b* n=6943. Source data are provided as a Source Data file.

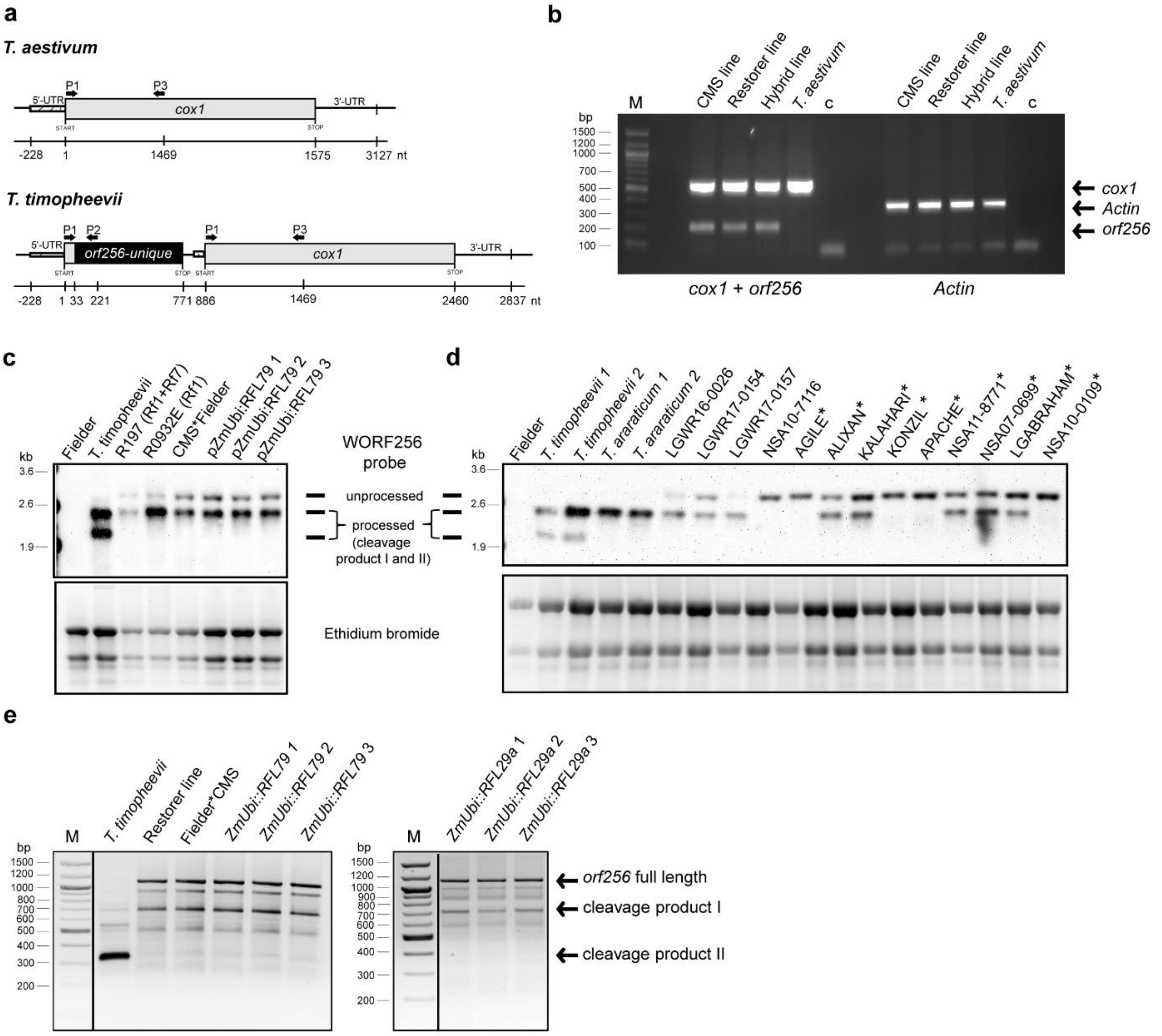

Supplementary Figure 4. Expression and processing of *orf256* in Fielder*CMS and *RFL79 and RFL29a* transformants. (**a**) Schematic overview of the *orf256* genomic structure. (**b**) RT-PCR analysis of the expression of *orf256* in different wheat genotypes. The experiment was performed three times and similar results were obtained. (**c**) Northern blot analysis of *orf256* processing in mitochondria of several wheat accessions. The probe used to detect *orf256* was prepared as described previously[1]. The experiment was performed twice and similar results were obtained. (**d**) Survey of available T-CMS accessions in regard to *orf256* processing. Several sterile lines including Alixan, Kalahari, Apache show processing of *orf256* at cleavage site I that does not correlate with the restoration of fertility. Cleavage site II was detected only in *T. timopheevii*. Asterisks indicate CMS lines. M - RNA Markers. The experiment was performed twice and similar results were obtained. (**e**) Mapping the 5'-ends of *orf256* RNA by 5'RACE analysis. Cleavage product I of *orf256* was amplified in CMS as well restorer lines. Cleavage product II was amplified only in *T. timopheevii*. The cleavage of *orf256* does not correlate with the restoration phenotype observed for the *RFL79 and RFL29a* transformants. The amplification products were obtained with Gene Specific Primer 1 (GSP1) listed in Supplementary Table 6. M - 100 bp DNA ladder. The experiment was performed three times and similar results were obtained. Source data underlying Supplementary Figure 4b-e are provided as a Source Data file.

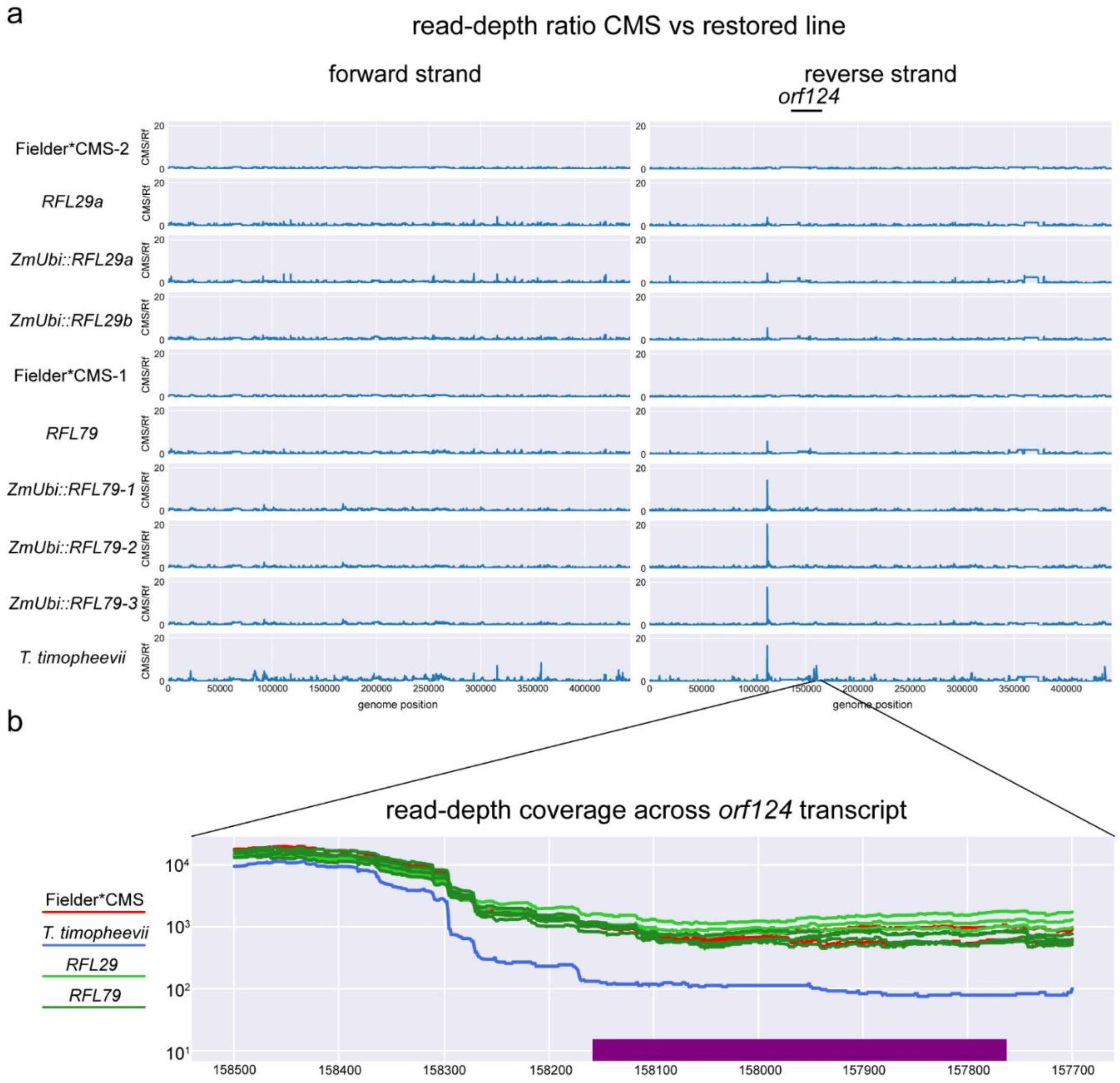

Supplementary Figure 5. Analysis of expression pattern of *orf124* between sterile and restored genotypes. (**a**) Ratio of strand-specific RNA-Seq coverage from Fielder*CMS lines (sterile) and restored (fertile) samples plotted across the *T. timopheevii* mitochondrial genome (NCBI accession number NC_022714.1) for forward and reverse strand is shown. The genomic region carrying *orf124* (reverse strand) is indicated at the top of the plots. (**b**) Normalised RNA-Seq coverage in the *orf124* region across the accessions. RNA-Seq counts and Python code used for plotting (**a**) and (**b**) are available from Dryad (https://doi.org/10.5061/dryad.6djh9w10d).

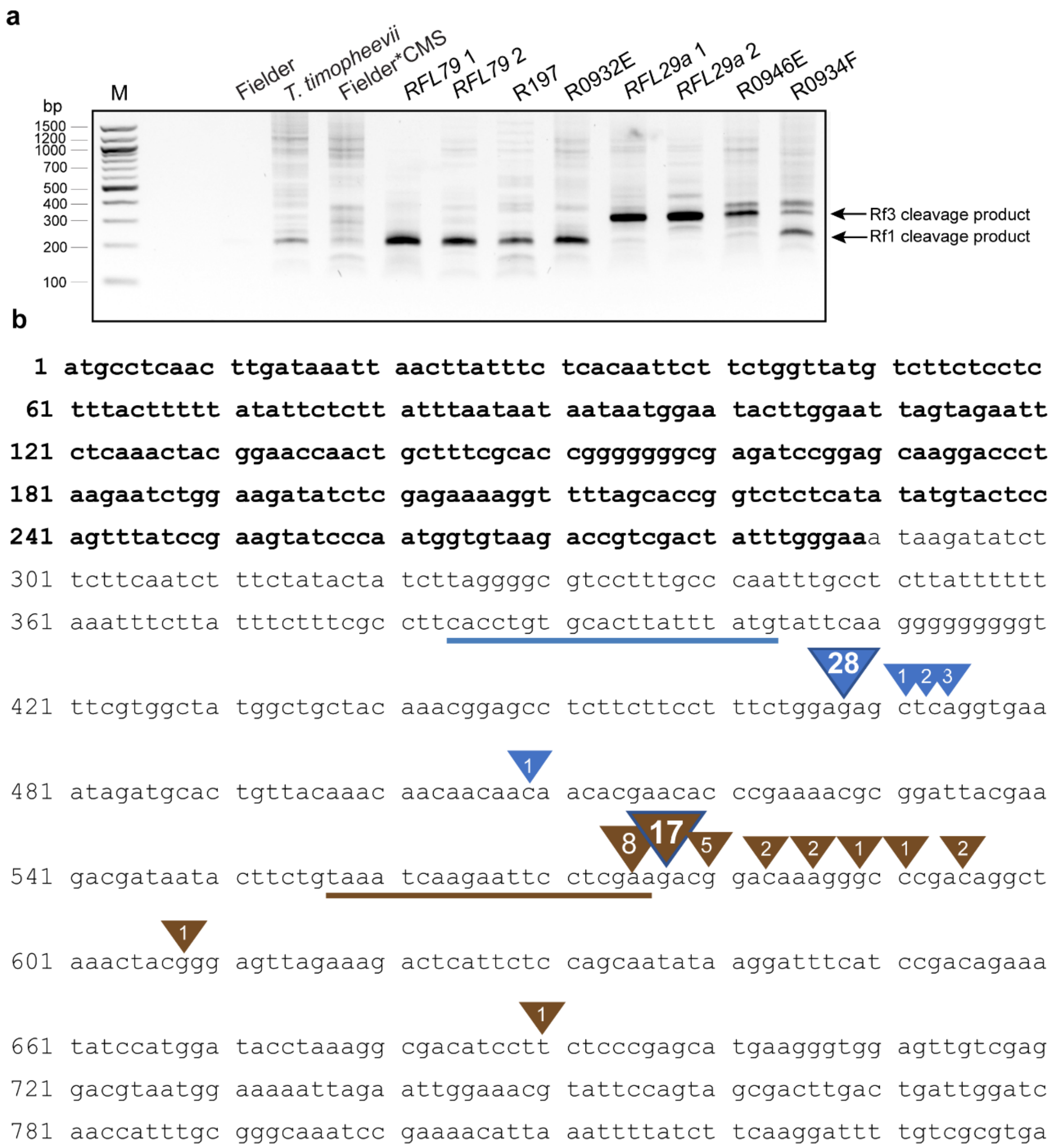

Supplementary Figure 6. Mapping cleavage sites of *orf279* RNA by 5'-RACE approach. (**a**) 5'-RACE reaction products separated on 1.5% agarose gel and stained with ethidium bromide. For amplification the Gene Specific Primer 1 (GSP1) listed in Supplemental Table 8 was used. M: 100 bp NEB ladder. The experiment was performed three times and similar results were obtained. (**b**) Position of mapped cleavage sites within *orf279* RNA identified by sequencing of the obtained 5'-RACE products. The sequence encoding the first 96 amino acid residues at the N-terminus of Orf279 corresponding to the ATP synthase subunit 8 encoded by the mitochondrial *atp8* gene is shown in bold. The blue and brown triangles point to cleavage sites identified in *RFL29a* and *RFL79* transformants, respectively. The numbers within the triangles indicate the number of clones sequenced to contain RNA starting at this position. The predicted binding sites for RFL29a and RFL79 are underlined in blue and brown, respectively. Source data underlying Supplementary Figure 6a are provided as a Source Data file.

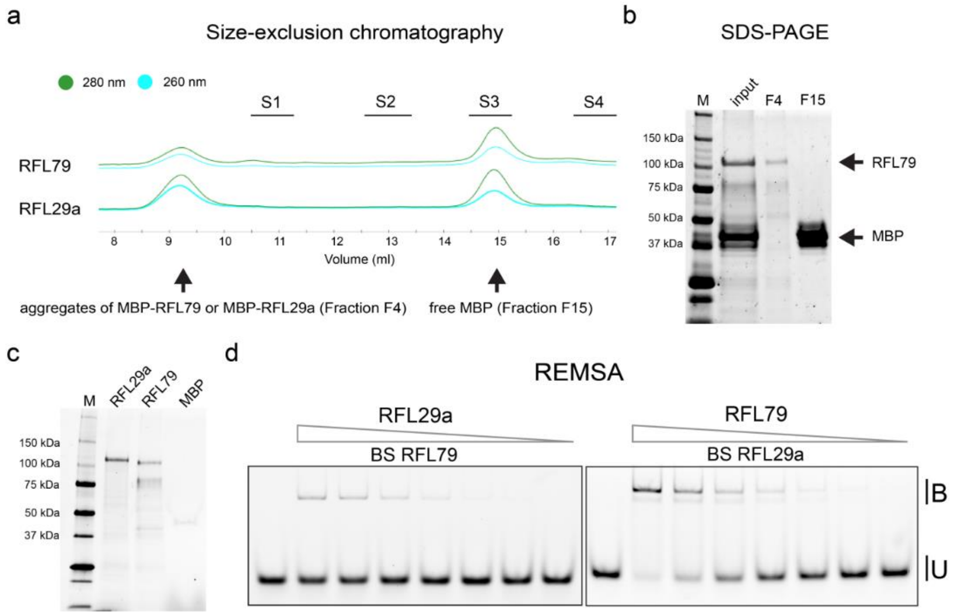

Supplementary Figure 7. *In vitro* RNA-binding assays with recombinant RFL29a and RFL79 proteins. The RFL29a and RFL79 proteins were expressed as N-terminal fusions with Maltose Binding Protein (MBP) and Histidine-tag (His-tag). The recombinant proteins were purified on Ni-NTA columns (Bio-Rad) by gravity flow. (**a**) To analyse the state of the recombinant proteins (ratio of soluble fraction vs aggregates), the purified proteins were subjected to size-exclusion chromatography (SEC) using an ENrich™ SEC 650 column (Bio-Rad) on an NGC Chromatography System (Bio-Rad). The size of separated proteins detected by 280 nm absorbance was estimated using size exclusion standards (Bio-Rad): thyroglobulin Mr 670,000 (S1), bovine γ-globulin Mr 158,000 (S2), chicken ovalbumin Mr 44,000 (S3), equine myoglobin Mr 17,000 (S4). The approximate retention of the standards is indicated. The SEC analysis revealed the presence of high-molecular weight protein aggregates of RFL29a or RFL79 eluting first followed by protein fractions with estimated molecular weight similar to free MBP. The experiment was performed once. (**b**) The fractions collected during the run with a BioFrac fraction collector (Bio-Rad) were separated on a Mini-PROTEAN TGX Stain-Free gel (Bio-Rad). SDS-PAGE confirmed that the protein aggregates seen during the SAC analysis represent recombinant RFL79 protein. Input - Ni-NTA purified RFL79 protein fraction before SEC analysis, F4 - fraction containing high-molecular weight aggregates, F15 - fraction containing the low molecular weight protein. M - Molecular mass standard. The experiment was performed once. (**c**) SDS-PAGE of protein fractions used in all REMSA experiments. The experiment was performed once. (**d**) To study the specificity of RNA binding, the purified RFL29a and RFL79 were incubated with each other's predicted RNA targets. Both proteins bind to the other's RNA target as shown by REMSA assay. Serial protein dilutions ranging from 1.8 µM to 28.1 nM were used for RFL29a and RFL79. The final concentration of the RNA probes was 1 nM. B = bound - RNA-protein complex, U = unbound - free RNA probe. On each gel, the left lane acts as a marker for unbound probe. The experiment was performed three times and similar results were obtained. Source data are provided as a Source Data file.

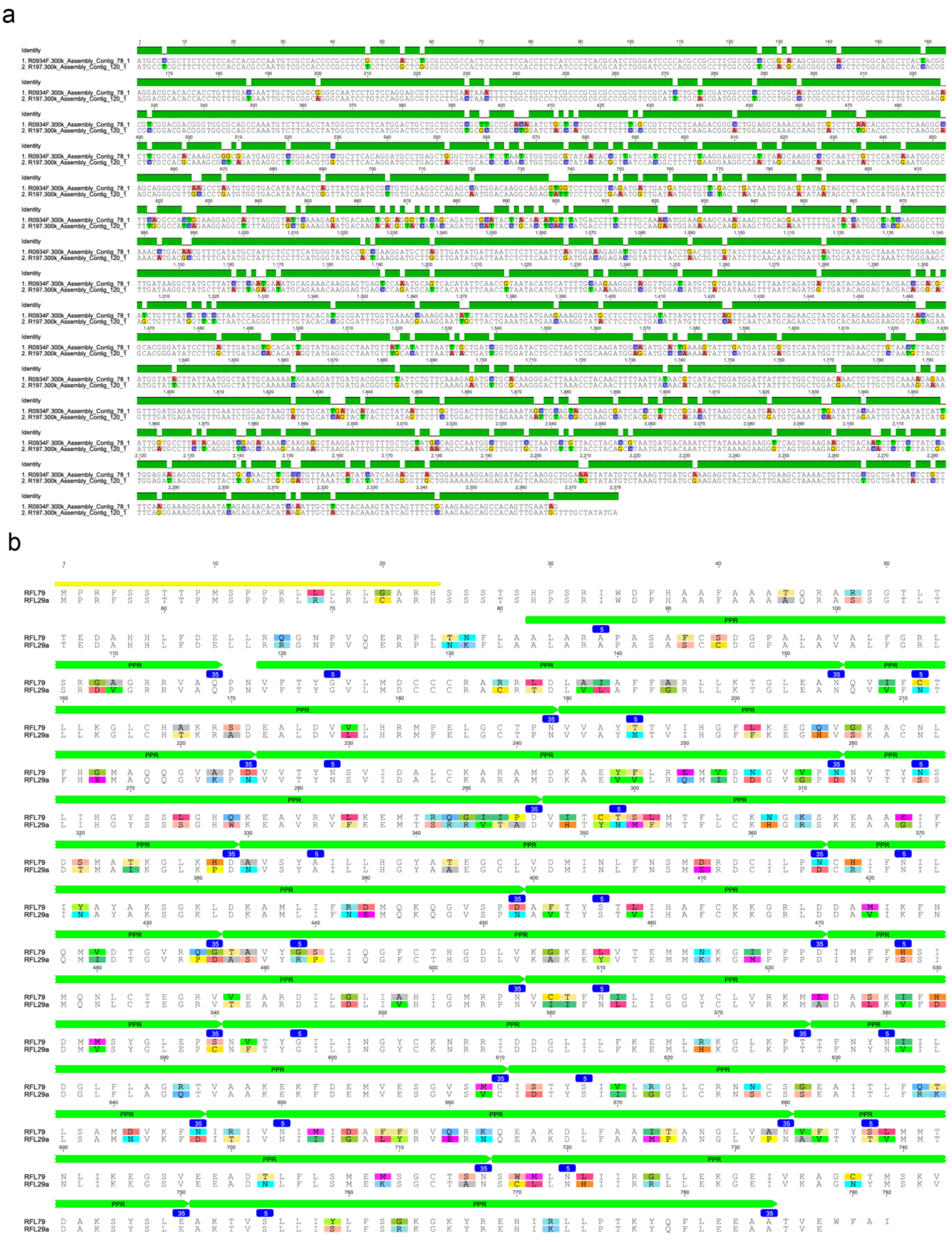

Supplementary Figure 8. Comparison between *RFL79* (*Rf1* candidate) and *RFL29a* (*Rf3*) candidate DNA sequences (**a**) and encoded protein sequences (**b**). The sequence of mitochondrial targeting peptide is highlighted by yellow box, PPR motifs by green boxes, and amino acid residues at position 5 and 35 of each PPR motif by blue boxes. Source data are provided as a Source Data file.

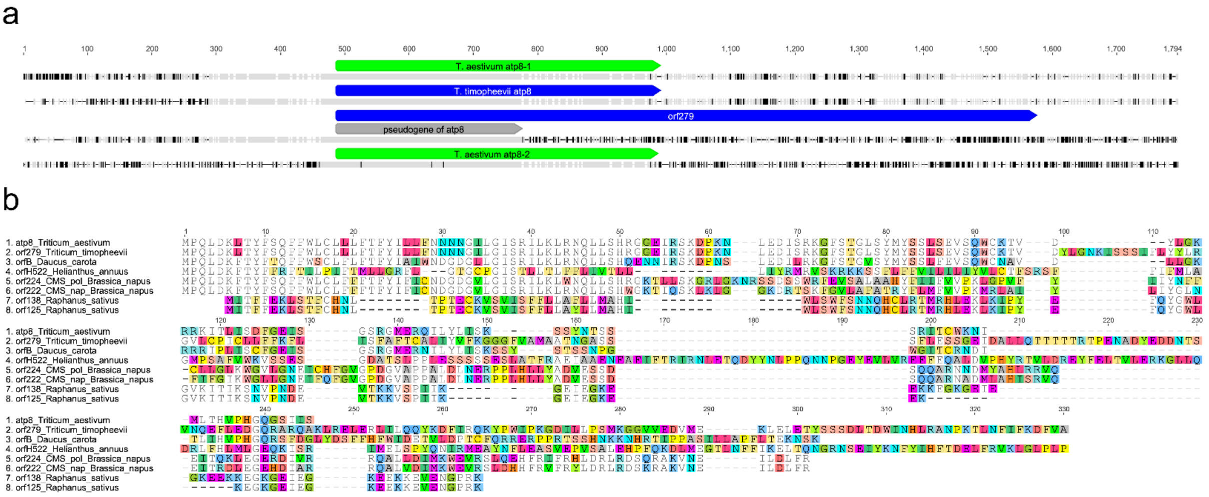

Supplementary Figure 9. *Orf279* as genetic basis of cytoplasmic male sterility in wheat T-CMS plants. (**a**) Alignment of the promoter and 5' UTR regions of *T. aestivum atp8-1* gene, *T. timopheevii* full-length *atp8* gene, *orf279*, and *T. aestivum atp8-2* gene (GenBank accessions numbers: AP013106/ NC_022714 and AP008982). (**b**) Phylogenetic relationships between chimeric *atp8*-orfs related to CMS in plants. Alignment was generated with Geneious (https://www.geneious.com) and tree with FastTree. The GenBank accession numbers for the CMS-genes are as follow: *orf138*[2] (P68513.1), *orf125*[3] (AB015327), *orfB*[4] (AB033490), *orf224* and *orf222*[5] (U10423 and U10428), *orfH522*[6] (CAA37614.1), *orf279* (this manuscript), *atp8* (NC_022714). Source data are provided as a Source Data file.

Supplementary Table 1. Markers identified in genetic screen as associated with fertility restoration conferred either by *Rf1* or *Rf3* locus in wheat.

Marker ID	Restorer	SNP	location	strand	START	STOP	Sequence
cfn0522096	Rf1	G/C	chr1A	[-]	14505133	14505203	ATGCAAAGTAGTACTCGTAGAGAGTTAACACAGAC[G/C]AGTGATTTATTGGGTGGTATTCTACTTGATATTTG
cfn0527067	Rf1	A/G	chr1A	[-]	15346055	15346425	GACAATATGATTCACCCTAGATCCTTCACCTTACA[A/G]TTCGAAAAAAATAAAAGAACAAAAGTAATTTGACA
cfn1249269	Rf3	A/G	chr1B	[+]	17780363	17780422	CGTTTAAAAGAACACAAATGTGGCCCTAGTGATCA[A/G]GTACACATATTTGTCACCTCTTTGAATCTTACTTA
BS00090770	Rf3	T/C	chr1B	[-]	20589170	20589229	TAGCCGTAGGTCGTAGCACATAGCCGTTTA[T/C]GTAATGCATAGTTGTCCGAAGGAATGTTTC

Supplementary Table 2. RFL genes identified in the *Rf1* and *Rf3* genomic regions in the IWGSC RefSeqv1.0 genome of Chinese Spring.

Interval	# RFL	Gene name	Location	START	STOP	Strand	Encoded protein	Protein length (aa)	Predicted location[7]	Comment
Rf1 (14.5-15.3 Mbp)	RFL77	TraesCS1A02G031600.1	chr1A	14577582	14579933	[+]	Pentatricopeptide repeat-containing protein (PPR)	784	m (0.853, mTP 20)	full-length
	RFL105*	TraesCS1A02G031700.1	chr1A	14584912	14587137	[+]	Pentatricopeptide repeat-containing protein (PPR)	741	m (0.845, mTP 23)	truncated
		TraesCS1A02G031800.1	chr1A	14589565	14594115	[-]	Protein kinase, putative			
		TraesCS1A02G031900.1	chr1A	14603494	14615692	[-]	Protein kinase, putative			
		TraesCS1A02G032000.1	chr1A	14744093	14748608	[-]	O-methyltransferase			
		TraesCS1A02G032100.1	chr1A	14790721	14791908	[-]	Chalcone synthase			
		TraesCS1A02G032200.1	chr1A	14799110	14800511	[-]	O-methyltransferase			
		TraesCS1A02G032300.1	chr1A	14838308	14839886	[-]	Chalcone synthase			
		TraesCS1A02G032400.1	chr1A	14857039	14858282	[-]	O-methyltransferase			
		TraesCS1A02G032500.1	chr1A	14988541	14988944	[+]	alpha/beta-Hydrolases superfamily protein			
		TraesCS1A02G032600.1	chr1A	15186553	15188366	[+]	Anthocyanidin synthase			
		TraesCS1A02G032600.2	chr1A	15177031	15177339	[+]	Anthocyanidin synthase			
		TraesCS1A02G032700.1	chr1A	15190979	15195959	[+]	Replication protein A 70 kDa DNA-binding subunit			
Rf3 (17.8-20.6 Mbp)	RFL60	TraesCS1B02G038200.1	chr1B	17894195	17896564	[-]	Pentatricopeptide repeat-containing protein (PPR)	790	m (0.910, mTP 25)	full-length
	RFL164	TraesCS1B02G038300.1	chr1B	17964601	17966241	[-]	Pentatricopeptide repeat-containing protein (PPR)	546	m (0.883, mTP 14) (pseudogene)	truncated
	RFL396	TraesCS1B02G038400.1	chr1B	18091427	18093253	[-]	Pentatricopeptide repeat-containing protein (PPR)	608	none (pseudogene)	truncated
	RFL29b	TraesCS1B02G038500.1	chr1B	18116277	18118649	[-]	Pentatricopeptide repeat-containing protein (PPR)	790	m (0.899, mTP 25)	full-length
	RFL252	TraesCS1B02G038600.1	chr1B	18363378	18364565	[-]	Pentatricopeptide repeat-containing protein (PPR)	396	none (pseudogene)	truncated
		TraesCS1B02G038700.1	chr1B	18385559	18387800	[-]	Nitrate transporter 1.1			
		TraesCS1B02G038800.1	chr1B	18419377	18423130	[-]	Protein kinase			
		TraesCS1B02G038900.1	chr1B	18570322	18571755	[-]	Cysteine protease			
		TraesCS1B02G039000.1	chr1B	18578571	18579939	[+]	Glycosyltransferase			
	RFL89	TraesCS1B02G039100.1	chr1B	18683381	18685732	[+]	Pentatricopeptide repeat-containing protein (PPR)	783	m (0.936, mTP 18)	full-length

	RFL58	TraesCS1B02G039200.1	chr1B	18867715	18870069	[+]	Pentatricopeptide repeat-containing protein (PPR)	784	m (0.874, mTP 19)	full-length
		TraesCS1B02G039300.1	chr1B	19018793	19020813	[+]	Oligopeptidase A			
		TraesCS1B02G039400.1	chr1B	19,025,038	19025620	[+]	Leucine-rich repeat protein kinase family protein			
		TraesCS1B02G039500.1	chr1B	19038922	19043415	[+]	Receptor protein kinase, putative			
		TraesCS1B02G039600.1	chr1B	19047105	19051706	[+]	Protein kinase, putative			
	RFL97f*	chr1B:R:19063406-19062072 TraesCS1B02G039625	chr1B	19061029	19063406	[-]	Pentatricopeptide repeat-containing protein (PPR)	445+363	m (0.946, mTP 16) (pseudogene)	truncated
	RFL97	TraesCS1B02G039625	chr1B	19073841	19076117	[-]	Pentatricopeptide repeat-containing protein (PPR)	759	m (0.715, mTP 22)	full-length
		TraesCS1B02G039800.1	chr1B	19217861	19232577	[-]	Receptor-like protein kinase			
		TraesCS1B02G039900.1	chr1B	19314295	19314714	[-]	Defensin			
		TraesCS1B02G040000.1	chr1B	19718626	19720046	[-]	Chalcone synthase			
		TraesCS1B02G040100.1	chr1B	19779389	19780576	[-]	Chalcone synthase			
		TraesCS1B02G040200.1	chr1B	19890445	19891967	[-]	Chalcone synthase			
		TraesCS1B02G040300.1	chr1B	19950229	19951416	[-]	Chalcone synthase			
		TraesCS1B02G040400.1	chr1B	20015264	20017794	[-]	O-methyltransferase-like protein			
		TraesCS1B02G040500.1	chr1B	20046035	20047787	[-]	Chalcone synthase			
		TraesCS1B02G040600.1	chr1B	20059838	20061082	[-]	O-methyltransferase			
		TraesCS1B02G040700.1	chr1B	20167177	20169022	[+]	Anthocyanidin synthase			
		TraesCS1B02G040800.1	chr1B	20172786	20177655	[+]	Replication protein A 70 kDa DNA-binding subunit			

Supplementary Table 3. Summary of plant genomes used in the study.

No.	Tribe	Species	Cultivar	Website	References	Identified RF-like PPR proteins
1	Triticeae	*Aegilops tauschii (1)*	AL8/78	http://plants.ensembl.org/Aegilops_tauschii/Info/Index	8	46
2		*Aegilops tauschii (2)*		http://wheat-urgi.versailles.inra.fr/Seq-Repository/Assemblies	8	31
3		*Aegilops speltoides*		http://wheat-urgi.versailles.inra.fr/Seq-Repository/Assemblies	9	20
4		*Aegilops sharonensis*		http://wheat-urgi.versailles.inra.fr/Seq-Repository/Assemblies	9	32
5		*Triticum aestivum*		http://plants.ensembl.org/Triticum_aestivum/Info/Index	9	98
6		*Triticum durum*	cv. Cappeli	http://wheat-urgi.versailles.inra.fr/Seq-Repository/Assemblies	9	30
7		*Triticum durum*	cv. Strongfiled	http://wheat-urgi.versailles.inra.fr/Seq-Repository/Assemblies	9	30
8		*Triticum monococcum*		http://wheat-urgi.versailles.inra.fr/Seq-Repository/Assemblies	10	12
9		*Triticum turgidum*		http://www.ncbi.nlm.nih.gov/bioproject/PRJNA191054	11	25
10		*Triticum urartu*		http://archive.gramene.org/Triticum_urartu/Info/Annotation/	12	24
11		*Hordeum vulgare*	Barke	http://pgsb.helmholtz-muenchen.de/plant/barley/index.jsp	13	13
12		*Hordeum vulgare*	Morex	http://pgsb.helmholtz-muenchen.de/plant/barley/index.jsp	13	12
13		*Hordeum vulgare*	Bowman	http://pgsb.helmholtz-muenchen.de/plant/barley/index.jsp	13	13
14		*Hordeum vulgare*	var. distichum	http://plants.ensembl.org/Hordeum_vulgare/Info/Index	13	0
15		*Lolium perenne*	P226/135/1	http://www.ncbi.nlm.nih.gov/nuccore/GAYX00000000	15	2
16		*Secale cereale*		http://pgsb.helmholtz-muenchen.de/plant/rye/index.jsp	15	0
17	Oryzeae	*Oryza sativa*		http://phytozome.jgi.doe.gov/pz/portal.html#!bulk?org=Org_Osativa	16	14
18		*Oryza sativa japonica*		http://plants.ensembl.org/Oryza_sativa/Info/Index	17	14
19		*Oryza indica*	Nipponbare	http://plants.ensembl.org/Oryza_indica/Info/Index	18	18
20		*Oryza sativa Nipponbare*		http://rapdb.dna.affrc.go.jp/index.html	17	14
21		rice cultivar 'Kasalath'	Kasalath	http://rapdb.dna.affrc.go.jp/index.html	18	11
22		*Oryza barthii*		http://plants.ensembl.org/Oryza_barthii/Info/Index	19	13
23		*Oryza brachyantha*		http://plants.ensembl.org/Oryza_brachyantha/Info/Index	20	4
24		*Oryza glaberrima*		http://plants.ensembl.org/Oryza_glaberrima/Info/Index	21	2
25		*Oryza glumaepatula*		http://plants.ensembl.org/Oryza_glumaepatula/Info/Index	19	12
26		*Oryza meridionalis*		http://plants.ensembl.org/Oryza_meridionalis/Info/Index	19	15
27		*Oryza nivara*		http://plants.ensembl.org/Oryza_nivara/Info/Index	19	13
28		*Oryza punctata*		http://plants.ensembl.org/Oryza_punctata/Info/Index	19	7
29		*Oryza rufipogon*		http://plants.ensembl.org/Oryza_rufipogon/Info/Index	19	12
30	Brachypodieae	*Brachypodium distachyon*		http://phytozome.jgi.doe.gov/pz/portal.html#!bulk?org=Org_Bdistachyon	22	11

31	Eragrostideae	*Eragrostis tef*		http://www.tef-research.org/index.html	[23]	33
32	Poniceae	*Setaria italica*		http://phytozome.jgi.doe.gov/pz/portal.html#!bulk?org=Org_Sitalica	[24]	15
33	Andropogoneae	*Sorghum bicolor*	BTx623	http://plants.ensembl.org/Sorghum_bicolor/Info/Index	[25]	20
34	Andropogoneae	*Sorghum bicolor*	BTx623	http://www.ncbi.nlm.nih.gov/	[25]	11
35	Andropogoneae	*Zea mays*	B73	http://plants.ensembl.org/Zea_mays/Info/Index	[26]	6
					Total	633
				Reference RFLs	[27]	49
				Sorghum WGS data sets	[28]	517
					Total	1199

Supplementary Table 4. Summary of the RFL capture experiment.

#	Restoration status	Accession name	Number of assembled contigs composed of > 100 reads	Number of identified RFL ORFs >210 aa*	Number of orthologous groups with at least one RFL from the accession	Number of RFL ORFs > 350 aa assigned to orthologous groups
1	weak Rf3 restorer	Chinese Spring	204	216	205	161
2	maintainer	Anapurna	211	221	202	156
3	maintainer	Fielder	231	223	212	138
4	*Rf1*	R197	219	241	219	174
5	*Rf1*	R0932E	221	245	221	183
6	*Rf3*	R0946E	239	262	237	171
7	*Rf3+RF1*	R0934F	223	237	215	174
8	*Rf3*	Primepi	226	234	215	162
9	*Rf1*	*Triticum timopheevii*	138	143	129	114
	Total		1912	2022	397 (non-redundant)	1433

Supplementary Table 5. Selection of candidate RFL groups based on restoring status of analysed wheat accessions and location within a given interval.

Selection of candidate RFL groups based on restoring status of analysed wheat accessions and location within the *Rf1* interval.

**RFL gene**	**Protein size (aa)**	**Gene located within *Rf1* interval (*in silico* mapping with tblastn)**	**Gene located within *Rf1* mapping interval (genetic mapping (nb of markers))**	**Restoring genotype**							
				**Maintainer**		***Rf1* restorer**		***Rf3* restorer**		***Rf1* + *Rf3***	***Rf1* restorer**
				**Chinese Spring**	**Anapurna**	**R197**	**R0932E**	**R0946E**	**Primepi**	**R0934F**	***Triticum timopheevii***
RFL1	988	no	n.a.	0	0	1*	1	0	0	0	0
RFL56	804	no	n.a.	0	0	1	1*	0	0	0	1
RFL59	813	no	n.a.	0	0	1	2	0	0	0	1
RFL73	813	no	n.a.	0	0	1	1	0	0	0	1
RFL74	813	no	n.a.	0	0	1	1	0	0	1	0
RFL79	808	no	yes (4)	0	0	1	1	0	0	1	1
RFL93	775	no	no	0	0	1	1	0	0	0	0
RFL104	757	yes	yes (4)	0	0	1	1	0	0	1	1
RFL129*	693	no	no	0	0	1	1	0	0	1	0
RFL185*	524	yes	yes (4)	0	0	1	1	0	0	1	1
RFL268*	382	yes	yes (4)	0	0	1	1	0	0	1	1

* - non-functional sequence truncated or disrupted by a frameshift.

Selection of candidate RFL groups based on restoring status of analysed wheat accessions and location within the *Rf3* interval.

**RFL gene**	**Protein size (aa)**	**Gene located within *Rf3* interval (*in silico* mapping with tblastn)**	**Gene located within *Rf3* mapping interval (genetic mapping (nb of markers))**	**Restoring genotype**								
				**weak *Rf3* restorer line**	**Maintainer**	***Rf1* restorer**		***Rf3* restorer**		***Rf1* + *Rf3***	***Rf1* restorer**	**Maintainer**
				**Chinese Spring**	**Anapurna**	**R197**	**R0932E**	**R0946E**	**Primepi**	**R0934F**	***Triticum timopheevii***	**Fielder**
RFL67	820	yes	yes	0	0	0	0	1	1	1	0	0
RFL89	801	no	yes (7)	1	0	0	0	1	1	1	0	1
RFL140*	637	no	yes (4)	0	0	0	0	1	1	1	0	0
RFL164*	391	yes	yes (6)	1	0	0	0	1	1	1	0	0
RFL166*	568	no	yes (2)	0	0	0	0	1*	1*	1*	0	0
RFL252*	396	no	yes (9)	1	0	0	0	1	1	1	0	0
RFL28	824	no	no	1*	1*	1*	1*	1	1	1	0	0
RFL29	790	yes	yes (7)	1	0	0	0	1	1	1	0	1*
RFL170	560	no	no	1*	1*	1*	0	1	1	1	0	1

* - non-functional sequence truncated or disrupted by a frameshift

Supplementary Table 6. List of oligos used in the study.

Oligo Name	Experiment	Sequence 5’->3’	Reference
Ta_Actin_F	qRT-PCR	GCCACACTGTTCCAATCTATGA	
Ta_Actin_R	qRT-PCR	TGATGGAATTGTATGTCGCTTC	
P1 (*orf256* + *coxI*)	qRT-PCR	ATGACAAATATGGTTCGATGGC	
P2 (*orf256*)	qRT-PCR	GCTTGGGGATCCTGAATC	
P3 (*coxI*)	qRT-PCR	GCTGTCACTAGAACGGACC	
orf256_GSP1	5’-RACE	GATTACGCCAAGCTTAAAACAGATCCTCCCTCATTCTCCCGCAGA	
orf279_GSP1	5’-RACE	GATTACGCCAAGCTTCATTACGTCCTCGACAACTCCACC	
RFL29a_attB1_f	REMSA	GGGGACAAGTTTGTACAAAAAAGCAGGCTTCATGCCCCGCTTCTCCTCCAC	
RFL29a_attB2_r	REMSA	GGGGACCACTTTGTACAAGAAAGCTGGGTCCTATTCAACTGTGGCTGCTTCTTCCAGA	
RFL79a_attB1_f	REMSA	GGGGACCACTTTGTACAAGAAAGCTGGGTCTCATATAGCAAACCATTCAACTGTGGCTG C	
RFL79a_attB2_r	REMSA	GGGGACAAGTTTGTACAAAAAAGCAGGCTTCATGTTCTGCAGCGATGGCCCT	
BS RFL29a	REMSA	CUUCACCUGUGCACUUAUUUAUGUAU	
BS RFL79	REMSA	CUGUAAAUCAAGAAUUCCUCGAAGA	
BS orf256	REMSA	CCACUAGCAGGUUUACUGCUUUCU	
WORF256_211_806_for	Nothern Blot	ATCCCCAAGCTCTAGCTCATTTAG	[1]
WORF256_211_806_rev	Nothern Blot	GGGGGCTGGAAGAGAAAAGAAT	[1]
RFL capture_qPCR_for	To confirm untargeted sequence depletion (chloroplas t genome sequence)	TTTGGTTTCAAAGCCCTACG	
RFL capture_qPCR_rev	To confirm untargeted sequence depletion	AACTTGGATACCATGAGGCG	

	(chloroplast genome sequence)		
ShBar_for	Genotyping of transgenic plants	GCACCATCGTCAACCACTACA	
ShBar_rev	Genotyping of transgenic plants	GTCCACTCCTGCGGTTCCT	
ShBar_TaqMan probe	Genotyping of transgenic plants	FAM-CACGGTCAACTTCCGTAC-MGB-NFQ	
GaMyb_for	Genotyping of transgenic plants	GATCCGAATAGCTGGCTCAAGTAT	
GaMyb_rev	Genotyping of transgenic plants	GGAGACTGCAGGTAGGGATCAAC	
GaMyb_TaqMan probe	Genotyping of transgenic plants	5’VIC-CGTGGCTCCTGCGATGCAGC-TAMRA	

Supplementary references

1. Song, J. S. & Hedgcoth, C. Influence of nuclear background on transcription of a chimeric gene (*orf256*) and *coxI* in fertile and cytoplasmic male-sterile wheats. *Genome* **37**, 203-209 (1994).
2. Bonhomme, S., Budar, F., Ferault, M. & Pelletier, G. A 2.5 kb NcoI fragment of Ogura radish mitochondrial DNA is correlated with cytoplasmic male-sterility in Brassica cybrids. *Curr Genet* **19**, 121-127 (1991).
3. Iwabuchi, M., Koizuka, N., Fujimoto, H., Sakai, T. & Imamura, J. Identification and expression of the kosena radish (*Raphanus sativus* cv. Kosena) homologue of the ogura radish CMS-associated gene, orf138. *Plant Molecular Biology* **39**, 183-188 (1999).
4. Nakajima, Y., Yamamoto, T., Muranaka, T. & Oeda, K. A novel orfB-related gene of carrot mitochondrial genomes that is associated with homeotic cytoplasmic male sterility (CMS). *Plant Molecular Biology* **46**, 99-107 (2001).
5. LHomme, Y., Stahl, R. J., Li, X. Q., Hameed, A. & Brown, G. G. Brassica nap cytoplasmic male sterility is associated with expression of a mtDNA region containing a chimeric gene similar to the pol CMS-associated orf224 gene. *Curr Genet* **31**, 325-335 (1997).
6. Kohler, R. H., Horn, R., Lossl, A. & Zetsche, K. Cytoplasmic male-sterility in sunflower is correlated with the co-transcription of a new open reading frame with the *Atpa* Gene. *Mol Gen Genet* **227**, 369-376 (1991).
7. Emanuelsson, O., Nielsen, H., Brunak, S. & von Heijne, G. Predicting subcellular localization of proteins based on their N-terminal amino acid sequence. *J Mol Biol* **300**, 1005-1016 (2000).
8. Jia, J. *et al.* Aegilops tauschii draft genome sequence reveals a gene repertoire for wheat adaptation. *Nature* **496**, 91-95 (2013).
9. Mayer, K. F. X. *et al.* A chromosome-based draft sequence of the hexaploid bread wheat (*Triticum aestivum*) genome. *Science* **345** (2014).
10. Brenchley, R. *et al.* Analysis of the bread wheat genome using whole-genome shotgun sequencing. *Nature* **491**, 705-710 (2012).
11. Krasileva, K. V. *et al.* Separating homeologs by phasing in the tetraploid wheat transcriptome. *Genome Biol* **14**, R66 (2013).
12. Ling, H. Q. *et al.* Draft genome of the wheat A-genome progenitor *Triticum urartu*. *Nature* **496**, 87-90 (2013).
13. Mayer, K. F. X. *et al.* A physical, genetic and functional sequence assembly of the barley genome. *Nature* **491**, 711-716 (2012).
14. Farrell, J. D., Byrne, S., Paina, C. & Asp, T. *De novo* assembly of the perennial ryegrass transcriptome using an RNA-Seq strategy. *PLoS ONE* **9**, e103567 (2014).
15. Martis, M. M. *et al.* Reticulate evolution of the rye genome. *Plant Cell* **25**, 3685-3698 (2013).
16. Ouyang, S. *et al.* The TIGR Rice Genome Annotation Resource: Improvements and new features. *Nucleic Acids Res* **35**, D883-D887 (2007).
17. Kawahara, Y. *et al.* Improvement of the *Oryza sativa* Nipponbare reference genome using next generation sequence and optical map data. *Rice* **6**,4 (2013).
18. Sakai, H. *et al.* Rice Annotation Project Database (RAP-DB): an integrative and interactive database for rice genomics. *Plant Cell Physiol* **54**, e6 (2013).
19. Jacquemin, J., Bhatia, D., Singh, K. & Wing, R. A. The International Oryza Map Alignment Project: development of a genus-wide comparative genomics platform to help solve the 9 billion-people question. *Current Opinion in Plant Biology* **16**, 147-156 (2013).

20. Chen, J. F. *et al.* Whole-genome sequencing of *Oryza brachyantha* reveals mechanisms underlying *Oryza* genome evolution. *Nature Commun* **4**, 1595 (2013).
21. Wang, M. *et al.* The genome sequence of African rice (*Oryza glaberrima*) and evidence for independent domestication. *Nat Genet* **46**, 982-988 (2014).
22. The International Brachypodium Initiative. Genome sequencing and analysis of the model grass *Brachypodium distachyon*. *Nature* **463**, 763-768 (2010).
23. Cannarozzi, G. *et al.* Genome and transcriptome sequencing identifies breeding targets in the orphan crop tef (*Eragrostis tef*). *BMC Genomics* **15**, doi:10.1186/1471-2164-15-581 (2014).
24. Bennetzen, J. L. *et al.* Reference genome sequence of the model plant Setaria. *Nat Biotechnol* **30**, 555–561 (2012).
25. Paterson, A. H. *et al.* The *Sorghum bicolor* genome and the diversification of grasses. *Nature* **457**, 551-556 (2009).
26. Schnable, P. S. *et al.* The B73 maize genome: complexity, diversity, and dynamics. *Science* **326**, 1112-1115 (2009).
27. Fujii, S., Bond, C. S. & Small, I. D. Selection patterns on restorer-like genes reveal a conflict between nuclear and mitochondrial genomes throughout angiosperm evolution. *P Natl Acad Sci USA* **108**, 1723-1728 (2011).
28. Mace, E. S. *et al.* Whole-genome sequencing reveals untapped genetic potential in Africa's indigenous cereal crop sorghum. *Nat Commun* **4**, 2320 (2013).
