# Supplementary material for: The genetic basis of cytoplasmic male sterility and fertility restoration in wheat
Source: Nat Commun. 2021 Feb 15;12:1036. doi: 10.1038/s41467-021-21225-0 (PMC7884431; doi:10.1038/s41467-021-21225-0)
Supplement: Supplementary file 3 — Reporting Summary [file 41467_2021_21225_MOESM3_ESM.pdf]

nature research

Corresponding author(s): Ian Small

Last updated by author(s): Jan 11, 2021

Reporting Summary

Nature Research wishes to improve the reproducibility of the work that we publish. This form provides structure for consistency and transparency in reporting. For further information on Nature Research policies, see Authors & Referees and the Editorial Policy Checklist .

Statistics

For all statistical analyses, confirm that the following items are present in the figure legend, table legend, main text, or Methods section.

n/a	Confirmed	
[ ]	[x]	The exact sample size (*n*) for each experimental group/condition, given as a discrete number and unit of measurement
[ ]	[x]	A statement on whether measurements were taken from distinct samples or whether the same sample was measured repeatedly
[x]	[ ]	The statistical test(s) used AND whether they are one- or two-sided<br>*Only common tests should be described solely by name; describe more complex techniques in the Methods section.*
[x]	[ ]	A description of all covariates tested
[x]	[ ]	A description of any assumptions or corrections, such as tests of normality and adjustment for multiple comparisons
[x]	[ ]	A full description of the statistical parameters including central tendency (e.g. means) or other basic estimates (e.g. regression coefficient) AND variation (e.g. standard deviation) or associated estimates of uncertainty (e.g. confidence intervals)
[x]	[ ]	For null hypothesis testing, the test statistic (e.g. *F*, *t*, *r*) with confidence intervals, effect sizes, degrees of freedom and *P* value noted<br>*Give P values as exact values whenever suitable.*
[x]	[ ]	For Bayesian analysis, information on the choice of priors and Markov chain Monte Carlo settings
[x]	[ ]	For hierarchical and complex designs, identification of the appropriate level for tests and full reporting of outcomes
[x]	[ ]	Estimates of effect sizes (e.g. Cohen's *d*, Pearson's *r*), indicating how they were calculated

Our web collection on statistics for biologists contains articles on many of the points above.

Software and code

Policy information about availability of computer code

Data collection	Primer Express v2.0; design of oligonucleotide primers<br>SDS Software v2.4; analysis of qPCR data<br>bbmap package v35.x; adapter-trimming and mapping of NGS reads to reference<br>Geneious 8; assembly of NGS sequence capture data<br>RepeatMasker v.3.3.0; identification and masking of repeated sequences<br>Salmon v1.3.0; pseudomapping of NGS reads to transcripts
Data analysis	TargetP; prediction of targeting signals<br>CD-HIT; clustering of sequences by similarity<br>EMBOSS 6.6.0; extraction of open reading frames<br>HMMER 3.1b; identification of motif profiles<br>OrthoMCL; clustering of sequences by putative orthology<br>TMHMM; prediction of transmembrane helices<br>analySIS getIT! software v5.2, image analysis software for microscopes with digital cameras

For manuscripts utilizing custom algorithms or software that are central to the research but not yet described in published literature, software must be made available to editors/reviewers. We strongly encourage code deposition in a community repository (e.g. GitHub). See the Nature Research guidelines for submitting code & software for further information.

Data

Policy information about availability of data

All manuscripts must include a data availability statement. This statement should provide the following information, where applicable:

- Accession codes, unique identifiers, or web links for publicly available datasets
- A list of figures that have associated raw data
- A description of any restrictions on data availability

Data supporting the findings of this work are available within the paper and its supplementary information files. A reporting summary for this article is available as a supplementary information file. The datasets and materials generated and analyzed during the current study are available from the corresponding author upon request. The sequencing data from this study is available from the National Center for Biotechnology Information Sequence Read Archive under the BioProject accession PRJNA595448 (https://www.ncbi.nlm.nih.gov/bioproject/?term=PRJNA595448https://www.ncbi.nlm.nih.gov/bioproject/?term=PRJNA595448) for the sequence capture data and accessions PRJNA595431 (https://www.ncbi.nlm.nih.gov/bioproject/?term=PRJNA595431https://www.ncbi.nlm.nih.gov/bioproject/?term=PRJNA595431) and PRJNA675907 (https://www.ncbi.nlm.nih.gov/bioproject/?term=PRJNA675907https://www.ncbi.nlm.nih.gov/bioproject/?term=PRJNA675907) for the RNA-Seq data. Assembled sequence capture data is available from GenBank (https://www.ncbi.nlm.nih.gov/genbank/) with the accession codes MT014021-MT015390. The source data underlying Figs 2a-c, 3b, 3d-f, 4b-e, 6c, 7b and Supplementary Figs S1, S3, S4b-e, S6a, S7b-d and 8 are provided as a Source Data file.

Field-specific reporting

Please select the one below that is the best fit for your research. If you are not sure, read the appropriate sections before making your selection.

☒ Life sciences ☐ Behavioural & social sciences ☐ Ecological, evolutionary & environmental sciences

For a reference copy of the document with all sections, see nature.com/documents/nr-reporting-summary-flat.pdf

Life sciences study design

All studies must disclose on these points even when the disclosure is negative.

Sample size	No sample-size calculations were done. Sample sizes were generally limited by the availability of the biological material (e.g. the number of transformation events). In general the results of our experiments are qualitative rather than quantitative and do not require statistical analysis.
Data exclusions	No data were excluded from the analyses
Replication	The replication of experiments was as described in the manuscript
Randomization	Wheat plants grown in the greenhouse or in the growth chambers were randomized in trays along with all required controls. Plant material collected for molecular analyses was harvested randomly.
Blinding	Blinding was not applicable because the study does not involve animals and/or human research participants.

Reporting for specific materials, systems and methods

We require information from authors about some types of materials, experimental systems and methods used in many studies. Here, indicate whether each material, system or method listed is relevant to your study. If you are not sure if a list item applies to your research, read the appropriate section before selecting a response.

Materials & experimental systems

n/a	Involved in the study	
☐	☒	Antibodies
☒	☐	Eukaryotic cell lines
☒	☐	Palaeontology
☒	☐	Animals and other organisms
☒	☐	Human research participants
☒	☐	Clinical data

Methods

n/a	Involved in the study	
☒	☐	ChIP-seq
☒	☐	Flow cytometry
☒	☐	MRI-based neuroimaging

Antibodies

Antibodies used	Custom-made anti-Orf279 antibody (GenScript), made by expressing part of the unique region of Orf279 encompassing amino acid residues 137-279 in E. coli and using it for immunisation of two rabbits.<br>anti-rabbit IgG (whole molecule) -Peroxidase antibody produced in goat. Sigma Aldrich #A0545 (Sigma-Aldrich)

Validation

The anti-Orf279 affinity was raised and validation performed by GenScript USA Inc and by the authors of this manuscript. A validation statement is provided by Sigma-Aldrich on the manufacturers website for the anti-rabbit IgG (whole molecule) - Peroxidase antibody.
