# Supplementary material for: The genetic basis of cytoplasmic male sterility and fertility restoration in wheat
Source: Nat Commun. 2021 Feb 15;12:1036. doi: 10.1038/s41467-021-21225-0 (PMC7884431; doi:10.1038/s41467-021-21225-0)
Supplement: Supplementary file 4 — Description of Additional Supplementary Files [file 41467_2021_21225_MOESM4_ESM.pdf]

Description of Additional Supplementary Files

Supplementary Data 1:

RFL groups inferred with CD-Hit. CD-Hit was used to group putatively orthologous sequences across accessions. A threshold of 96% sequence identity was chosen as it was found to be the best compromise between distinguishing paralogous sequences within each accession whilst grouping putatively orthologous sequences across accessions.

Supplementary Data 2:

Genotyping of the transgenic wheat plants. Transgene insertion in the genome was confirmed by PCR. Real-time PCR was carried out in 384-well reaction plates to estimate the number of transgenes integrated per plant. Two replicates were performed for each sample and a calibrator sample (known 1 copy of Bar control). An expression value of ~ 1 indicates single insertion of the transgene whereas a value higher than 1 indicates multiple insertions.
