# Supplementary material for: The genetic basis of cytoplasmic male sterility and fertility restoration in wheat
Source: Nat Commun. 2021 Feb 15;12:1036. doi: 10.1038/s41467-021-21225-0 (PMC7884431; doi:10.1038/s41467-021-21225-0)
Supplement: Supplementary file 2 — Peer Review File [file 41467_2021_21225_MOESM2_ESM.pdf]

Reviewers' comments:

Reviewer #1 (Remarks to the Author):

Melonek et reported the identification of two wheat fertility restorer genes Rf1 and Rf3 for T-CMS using a positional cloning strategy in combination with a selective sequence capture approach. Rf1 and Rf3 belong to RFL clade of the PPR family and share high sequence identity. Rf1 and Rf3 were shown to the orf279 transcript, resulting the cleavage of Orf279 RNA and the reduced accumulation of the Orf279 protein .Overall the identification of this wheat CMS-Rf system is a key step towards the establishment of hybrid wheat varieties.
Comments：
1)It will be helpful for readers if the authors can provide the evolutionary insights into the origin and diversification of Rf1, Rf3 and orf279 among different plant species.
2)In Fig 2b, I suggest to include pollen grain staining to highlight the normal pollen development at the maturation stage.
3)To clarify the function of genes, the detailed expression analysis of Rf1, Rf3 and orf279 using qRT-PCR, in situ hybridization as well as their role in tapetal PCD function during anther development is needed.

Reviewer #2 (Remarks to the Author):

A thorough study on a subject of great importance for global food production.

Bait capture

The filtering steps for the bait capture make sense. Avoiding repeats and high copy number regions is useful to avoid having to discard a large fraction of the sequencing reads that concern sequences from these regions and motifs. However, the description of the probes is too limited: ‘The final probes were synthetized as a probe pool’. Now the reader must look at the details of the SeqCap procedure of Roche to know what they look like. How long were the probes? How were they tiled? How many were used in total? I assume DNA probes were used (not RNA probes, as used in Arbor Biosciences’ myBaits). What is a ‘double capture’ protocol?

The assembly of the contigs with RFL genes was checked by the successful assembly of these genes from Chinese Spring and a comparison with the genome sequence.

Fine mapping

Finemapping was done using many F2 populations of reasonable size (135-246 individuals). Fine mapping using progenies is mentioned in Results and M&M, but only the final result is shown in Fig 1a and 1b. This was all done using the single bread wheat genome sequence available, which is not from the parents of the crosses. Minor question: Were any inconsistencies detected (between the genetic order and distance of the markers used and their physical position in the genome sequence)? Major question, referring to ‘The list of candidates was refined by excluding those that mapped outside the intervals established by the genetic mapping (Supplementary Table S6)’: were also some of the recombinant F3 plants used to verify the presence of the candidates and other orthologous RFL groups, using gene-specific PCR on the DNA from recombinant plants, next to the Rf3-containing Primepi and R0934F genotypes? This would verify the position and order of relevant genes, without relying on the correct assembly of the genome sequence altogether in this region with clusters of several RFL genes. True, the results presented later show that the assumption that the genome sequence is correct and representative for these plants, appears to be correct.

Reviewer #3 (Remarks to the Author):

The paper describes the identification of the genes underlying two fertility restoration loci in wheat. Armed with the knowledge of the restorer genes, the authors have been able to characterise the nature of the cytoplasmic male sterility in lines carry the T. timopheevi cytoplasmic male sterility. This work respects a significant advance in our understanding of CMS in wheat and should play a major role in advancing CMS as a method for developing a commercially viable hybrid system for wheat. A number of CMS sources have been explored for wheat but the T. timopheevi systems has been the most extensively studied. There are several alternative methods for fertility control including the use of gametocides and nuclear male sterility. Generally, the CMS approach has been preferred but the lack of efficient fertility restoration has proved limiting. Consequently, this study is important since it provides valuable knowledge on two major fertility restoration loci and opens the path for identifying addition loci and alleles.
Wheat is a strongly inbreeding species and in addition to the lack of a commercially viable fertility control system in wheat, there are a number of other factors which are likely to impede adoption of hybrid breeding. The authors do gloss over these limitations and the introductory paragraph presents an overoptimistic view.
The paper is well written, and the results are clearly presented. However, I have two concerns. First, the authors provide considerable detail on the molecular aspects of the work but almost no information on their phenotyping techniques. Since the measure that is reported is "% fertile events" it would appear that fertility is assessed purely on the basis of seed set, presumably using bagged heads. Simply counting seed set is a rather crude method for assessing fertility since only a small amount of pollen is required for fertilisation. It would be preferable to see pollen viability counts and ideally some description of pollen development in the control and transgenic lines. There is a comment that "No pollen was observed to be produced and no seed set by Fielder *CMS plants……", and Figure (2b) shows images of wheat heads with extruded anthers visible in RFL79 and possibly in ZmUbi::RFL79. However, the pictures are of such poor quality that have little value and could be replaced by some images of pollen development or pollen viability.
The second concern is the absence of any discussion of other fertility restoration loci or other CMS options for wheat. A viable hybrid seed production system for wheat will require efficient fertility restoration and neither of the genes identified restore full fertility and, presumably, still suffer from greatly reduced pollen viability. It is encouraging to see the result showing improved fertility in the pyramided transgenes and using strong promoters. This implies that there may be benefits in searching for alternative alleles at Rf1 and Rf3 and in examining the characteristics of other loci. Is there any indication that the CMS mechanisms elucidated in this paper, might apply to other wheat CMS option that have not been as well studied as the T. timopheevi system? It is understandable that the authors have not undertaken a study of alternative loci and alleles in the current paper but to only suggest further work on Rf1 and Rf3 as a route achieving full fertility, seems rather narrow.

Responses to reviewers' comments

The manuscript has been substantially revised. The major revisions include:

- The addition of molecular data for an additional *Rf3* line, R0946E (Figure 2); this line was added to the molecular analyses when we discovered that the *Rf3* line used as an example in the previous manuscript (R0932F) also carries an active *Rf1* allele
- RNA-Seq data at three stages of pollen development to show how *Rf* and mitochondrial gene expression vary across this critical stage (Figure 2)
- pollen-staining data showing more clearly the substantial effect of the restorer genes on pollen development and viability (Figs 2-4)
- the addition of a year's worth of additional fertility data on an extra generation of the fertility-restored wheat transformants (Figure 3)
- A demonstration that *Rf3* can restore fertility when driven by a tapetum-specific promoter, confirming that the major effect of this gene is in this cell layer (Figure 4)

We believe that this additional data satisfactorily responds to all the requests made by the reviewers, and that the results only strengthen our previous conclusions. We have also revised the text in several places as detailed in the responses to the reviewers. We thank the reviewers for their constructive reviews that have stimulated us to strengthen the manuscript. We apologise for the long wait, COVID-19 restrictions greatly hampered and delayed our experiments.

Reviewer #1

1) It will be helpful for readers if the authors can provide the evolutionary insights into the origin and diversification of Rf1, Rf3 and orf279 among different plant species.

The huge number of RFL genes in *Triticum* compared with relatives such as barley, rice and maize suggest that most of them arose relatively recently within the *Triticum/Aegilops* complex by gene duplications and recombination events. The *Rf1* and *Rf3* genes in *T. aestivum* presumably derive from *T. timopheevii* and possibly *Aegilops speltoides* (as B genome donor) respectively. *T. timopheevii* has not yet been sequenced, but we confirmed the presence of *Rf1* in this species in our *RFL* capture experiment. Neither *Rf1* nor *Rf3* are present in the only publicly available *Aegilops speltoides* genome sequence, but this may not be a complete assembly. Basically, there aren't yet enough high-quality sequenced genomes from *Triticum/Aegilops* to trace the complex evolution of these genes with any confidence. *orf279* appears to also be unique to the Triticeae and we have not found any related sequence elsewhere. We have added a sequence alignment (Supplementary Figure S9) that demonstrates that there is no obvious sequence similarity with any of the other *atp8*-associated CMS ORFs from other plant species.

2)In Fig 2b, I suggest to include pollen grain staining to highlight the normal pollen development at the maturation stage.

We have added pollen staining results to the figure (now Figure 3) as requested. We have also included pollen staining data in two new experiments we have added to the manuscript (in Figures 2 and 4). The pollen staining data fully confirms the fertility data we had previously reported.

3)To clarify the function of genes, the detailed expression analysis of Rf1, Rf3 and orf279 using qRT-PCR, in situ hybridization as well as their role in tapetal PCD function during anther development is needed.

We have added a new figure (Figure 2) that provides data on expression of all *RFL* genes in *Rf1*-containing, *Rf3*-containing and non-restoring genotypes obtained via RNA-Seq at three stages of pollen development. We don't think that in situ hybridisation is feasible for these genes given a) their very low expression level (< 1 transcript per million) and b) the presence of hundreds of very similar transcripts making cross-hybridisation an issue. Instead, to address tissue-specificity, we expressed the Rf3 candidate (RFL29a) with a tapetum-specific promoter (*ZmMac2*) and showed that this almost completely restores male fertility (new Figure 4). We have not examined PCD as the mechanism by which T-CMS causes sterility is irrelevant to its genetic control by *Rf1* and *Rf3* — these restorer genes prevent the accumulation of the CMS-inducing protein, not its effects. We agree that such a study would be interesting, but potentially a huge amount of work and out of the scope we intended for this research, which is to focus on the molecular action of Rf1 and Rf3.

Reviewer #2

How long were the probes? How were they tiled? How many were used in total? I assume DNA probes were used (not RNA probes, as used in Arbor Biosciences' myBaits). What is a 'double capture' protocol?

We have added more details of the RFL capture protocol by adding the following sentences to the Online Methods section:

"A set of 62,579 DNA probes ranging from 50 to 95 nucleotides were designed. On average, probes were tiled every 36 bp on the targets."

"and two consecutive rounds of sequence capture, referred to as the double capture protocol, as recommended by the manufacturer in case of limited cumulated size of the targets in the genome."

Minor question: Were any inconsistencies detected (between the genetic order and distance of the markers used and their physical position in the genome sequence)?

At the macro-synteny level (see graphs below for both loci) the order of the markers — determined by the recombinant blocks in our mapping populations — were consistent with the order and orientation of the scaffolds on the available reference genome.

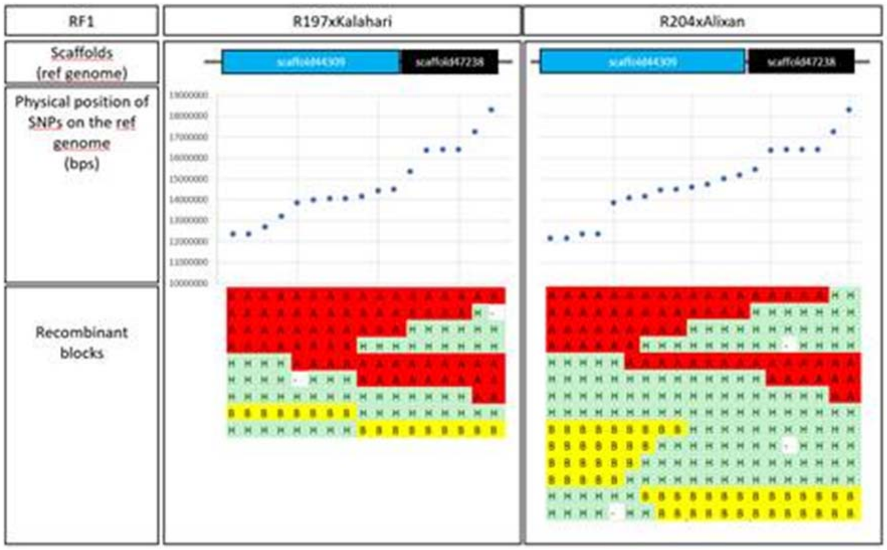

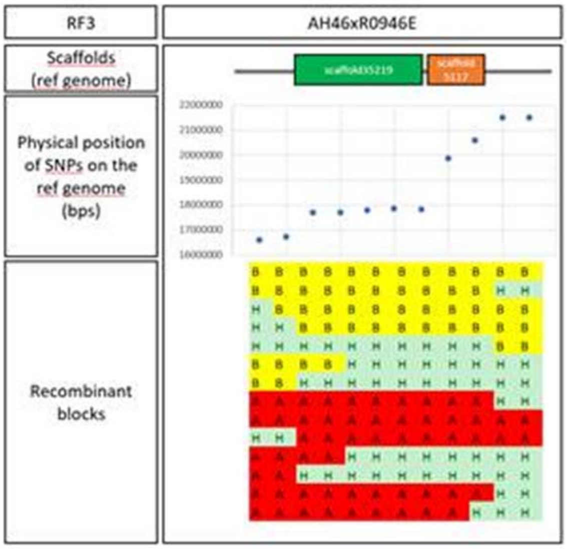

*SNP markers on the recombinant blocks (on F2 plants) are ordered following the physical position on the reference genome. A and B are homozygous genotypes for the sterile and fertile parent. H corresponds to a heterozygous genotype.

Major question, referring to 'The list of candidates was refined by excluding those that mapped outside the intervals established by the genetic mapping (Supplementary Table S6)': were also some of the recombinant F3 plants used to verify the presence of the candidates and other orthologous RFL groups, using gene-specific PCR on the DNA from recombinant plants, next to the Rf3-containing Primepi and R0934F genotypes? This would verify the position and order of relevant genes, without relying on the correct assembly of the genome sequence altogether in this region with clusters of several RFL genes. True, the results presented later show that the assumption that the genome sequence is correct and representative for these plants, appears to be correct.

No, we verified the genetic positions of candidate RFLs only in the populations that we retained for fine-mapping (for ex. see AH46xR0946E polymorphic SNP order versus IWGSCWGAV01 ref genome in answer to previous question). However, we have evidence that the parents used for fine-mapping are representative of those plants. First, all the markers within the locus (comprising specific

markers to the RFL families) have been genotyped on a diversity panel including all our parental lines (unpublished) and the molecular profiles of Primepii, R0934F, TJB155 and R0946E within the QTL interval are identical. Second, an extra sequencing experiment (unpublished) verified that the RFL sequences at the *Rf3* locus are identical for Primepii, R0934F, TJB155 and R0946E.

Reviewer #3 (Remarks to the Author):

Wheat is a strongly inbreeding species and in addition to the lack of a commercially viable fertility control system in wheat, there are a number of other factors which are likely to impede adoption of hybrid breeding. The authors do gloss over these limitations and the introductory paragraph presents an overoptimistic view.

We have extended the Discussion to acknowledge some of these 'other factors' that the reviewer refers to. We believe that the long-standing and continuing interest in this topic by many plant breeding companies amply illustrates that although there remain problems to be solved, these are not seen as insurmountable.

The paper is well written, and the results are clearly presented. However, I have two concerns. First, the authors provide considerable detail on the molecular aspects of the work but almost no information on their phenotyping techniques. Since the measure that is reported is "% fertile events" it would appear that fertility is assessed purely on the basis of seed set, presumably using bagged heads. Simply counting seed set is a rather crude method for assessing fertility since only a small amount of pollen is required for fertilisation. It would be preferable to see pollen viability counts and ideally some description of pollen development in the control and transgenic lines.

To address the reviewer's concerns we have added more detailed analyses of fertility including seed set per ear and per spikelet (Figure 3f and Supplemental Figure S3). In addition, we performed pollen viability stains and counts (also to address the comment of Reviewer #1). These results are included in the manuscript in the revised Figure 3.

There is a comment that "No pollen was observed to be produced and no seed set by Fielder *CMS plants……", and Figure (2b) shows images of wheat heads with extruded anthers visible in RFL79 and possibly in ZmUbi::RFL79. However, the pictures are of such poor quality that have little value and could be replaced by some images of pollen development or pollen viability.

We have added a new series of pictures of flowering heads taken with better resolution, but moved them to the supplementary data (Supplementary Figure S3) and, as suggested, replaced them in the main figure (now Figure 3) with images of stained pollen to better illustrate the contrast between the sterile and fertile plants. We have also added two new figures (Figures 2 and 4) that add new information on pollen development and expression of *Rf* genes in these wheat genotypes.

The second concern is the absence of any discussion of other fertility restoration loci or other CMS options for wheat. A viable hybrid seed production system for wheat will require efficient fertility restoration and neither of the genes identified restore full fertility and, presumably, still suffer from greatly reduced pollen viability.

A short "discussion of other fertility restoration loci or other CMS options for wheat" was added to the Discussion to better put our work into context. In the Fielder background we have used for our experiments, and under the growth conditions used, the pollen viability of the *Rf1*, *Rf3* and *Rf1Rf3* lines is actually very good, and in many cases not distinguishable from the fertile Fielder control.

It is encouraging to see the result showing improved fertility in the pyramided transgenes and using strong promoters. This implies that there may be benefits in searching for alternative alleles at Rf1 and Rf3 and in examining the characteristics of other loci. Is there any indication that the CMS mechanisms elucidated in this paper, might apply to other wheat CMS option that have not been as well studied as the T. timopheevi system? It is understandable that the authors have not undertaken a study of alternative loci and alleles in the current paper but to only suggest further work on Rf1 and Rf3 as a route achieving full fertility, seems rather narrow.

We do think that the new insights on the mechanism of CMS and fertility restoration elucidated in this paper will help in characterising other CMS systems in wheat. The approaches used in this study are certainly transferrable to other systems. Indeed, in future work we hope to study some of these other systems ourselves, in addition to pursuing the improvement of *Rf1*- and *Rf3*-based systems.

Reviewer #1 (Remarks to the Author):

I appreciate the efforts from the authors in revising this manuscript to address my comments.

Reviewer #2 (Remarks to the Author):

The authors have clarified and expanded the manuscript significantly. I am satisfied with the revisions.

Reviewer #3 (Remarks to the Author):

The reviewers highlighted several areas where the paper could be improved. In the revised version, the authors have addressed the issues raised. The additional information on methods, the assessment of pollen viability, the anther expression analysis and the revised discussion have significantly improved the paper.
This is a well-written and thorough study on an important topic and I have no remaining concerns.

POINT-BY-POINT RESPONSE TO THE REVIEWERS' COMMENTS

Manuscript: "**The genetic basis of cytoplasmic male sterility and fertility restoration in wheat**"

REVIEWERS' COMMENTS

Reviewer #1 (Remarks to the Author):

I appreciate the efforts from the authors in revising this manuscript to address my comments.

Reviewer #2 (Remarks to the Author):

The authors have clarified and expanded the manuscript significantly. I am satisfied with the revisions.

Reviewer #3 (Remarks to the Author):

The reviewers highlighted several areas where the paper could be improved. In the revised version, the authors have addressed the issues raised. The additional information on methods, the assessment of pollen viability, the anther expression analysis and the revised discussion have significantly improved the paper.
This is a well-written and thorough study on an important topic and I have no remaining concerns.

AUTHOR'S RESPONSE:

We greatly appreciate the time and effort of the three Reviewers spent on reading and reviewing of our work. Their comments and suggestions have been very helpful in improving the manuscript. Thank you!
